# Cardiometabolic Profiles of Oral and Subcutaneous Glucagon‐Like Peptide‐1 Receptor Mono‐Agonists in Adults With Overweight or Obesity: A Systematic Review and Network Meta‐Analysis

**DOI:** 10.1111/dom.70742

**Published:** 2026-04-16

**Authors:** Ying Lu, Jiajie Chen, Yuqing Guo, Hao Ding, Yu‐Lun Liu, Michelle A. Van Name, Mona Sharifi, Yuan Lu, Yong Chen

**Affiliations:** ^1^ Ividence Inc Newark Delaware USA; ^2^ UT Southwestern Medical Center, Peter O'Donnell Jr. School of Public Health Dallas Texas USA; ^3^ Pediatric Endocrinology, Department of Pediatrics Yale School of Medicine New Haven Connecticut USA; ^4^ Section of General Pediatrics, Department of Pediatrics Yale School of Medicine New Haven Connecticut USA; ^5^ Section of Cardiovascular Medicine, Department of Internal Medicine Yale School of Medicine New Haven Connecticut USA; ^6^ Center for Outcomes Research and Evaluation, Yale New Haven Hospital New Haven Connecticut USA; ^7^ The Center for Health AI and Synthesis of Evidence (CHASE) University of Pennsylvania Philadelphia Pennsylvania USA

**Keywords:** antiobesity drug, GLP‐1 analogue, network meta‐analysis, weight management

## Abstract

**Aims:**

To characterize the cardiometabolic profiles of oral and subcutaneous glucagon‐like peptide‐1 (GLP‐1) receptor mono‐agonists in adults with overweight or obesity, with or without type 2 diabetes (T2D), using network meta‐analysis (NMA).

**Materials and Methods:**

PubMed, Embase and CENTRAL were searched (January 2014–November 2025) for randomized controlled trials (RCTs) evaluating GLP‐1 receptor mono‐agonists (semaglutide, liraglutide and orforglipron) in adults with overweight or obesity. The primary outcome was the cardiometabolic efficacy index (CEI), a ranking‐based composite (0 to 1) summarizing performance across seven cardiometabolic endpoints: total body weight loss percentage, triglycerides, HDL cholesterol‐C, LDL‐C, waist circumference, HbA1c and systolic blood pressure. Secondary outcomes included treatment effects for each individual CEI component.

**Results:**

Nineteen RCTs (*N* = 13 117) were analysed. Semaglutide 7.2 mg achieved the highest CEI (0.86), followed by orforglipron 36 mg (bioequivalent to Foundayo 17.2 mg tablet) (0.68) and semaglutide 2.4 mg (0.66), all exhibiting placebo‐adjusted weight reductions ≥ 10%. CEI rankings were generally consistent across T2D and non‐T2D subgroups. Among oral formulations in non‐T2D adults, OFG 36 mg showed a CEI comparable to oral semaglutide 25 mg (0.67 vs 0.63).

**Conclusions:**

Higher‐dose GLP‐1 receptor mono‐agonists, particularly semaglutide 7.2 mg and orforglipron 36 mg (Foundayo 17.2 mg tablet), demonstrated the most consistent multidimensional cardiometabolic improvements, although domain‐specific differences were observed across agents.

## 1 Background

Glucagon‐like peptide‐1 (GLP‐1) receptor agonists have transformed obesity pharmacotherapy, producing substantial weight reduction while improving glycemia, blood pressure and lipid profiles [1]. These effects position GLP‐1 receptor agonists (GLP‐1RAs) as cardiometabolic therapies rather than weight‐loss agents alone. Despite multiple formulations and doses, direct head‐to‐head comparisons remain limited [2, 3], and treatment decisions increasingly require considering multidimensional cardiometabolic effects beyond weight loss.

Treatment options have expanded rapidly. Several GLP‐1RA agents, formulations and doses are now in clinical use or late‐stage development, including oral options such as semaglutide and the non‐peptide agent orforglipron. While subcutaneous agents have demonstrated durable benefits in randomized trials, parenteral administration may limit adherence. Oral formulations may improve acceptability and persistence, further expanding therapeutic choice.

However, comparative evidence remains limited. Existing network meta‐analyses (NMAs) have primarily focused on weight loss, potentially overlooking broader cardiometabolic effects [4, 5]. We therefore conducted a systematic review and NMA of RCTs evaluating oral and subcutaneous GLP‐1 receptor mono‐agonists in adults with overweight or obesity, with or without type 2 diabetes (T2D). Treatment effects across seven cardiometabolic risk factors were summarized using a cardiometabolic efficacy index (CEI), a ranking‐based composite metric designed to characterize multidimensional cardiometabolic profiles across agents, formulations and doses.

## 2 Materials and Methods

### 2.1 Study Design and Data Source

This systematic review and NMA followed PRISMA 2020 guidelines and was prospectively registered in PROSPERO (CRD420251238152). PubMed, Embase and the Cochrane Central Register of Controlled Trials (CENTRAL) were searched from January 2014 through November 2025 for RCTs evaluating GLP‐1 receptor agonists for weight management (search strategies in Table S1).

### 2.2 Search Strategy and Study Selection

Eligible RCTs enrolled adults with overweight (BMI ≥ 27 kg/m^2^ with comorbidity) or obesity (BMI ≥ 30 kg/m^2^), included ≥ 200 participants and ≥ 44 weeks of follow‐up, compared a GLP‐1 receptor mono‐agonist with placebo or an active comparator, and reported at least one prespecified outcome. Only RCTs were included to minimize confounding and preserve internal validity and the transitivity assumption for NMA [6, 7].

To reflect the current RCT evidence base and support the transitivity assumption for NMA [8], analyses were restricted to GLP‐1 receptor mono‐agonists that were FDA‐approved or under regulatory review for weight management, reflecting established dosing regimens rather than exact pharmacologic equivalence: subcutaneous semaglutide (1.7, 2.4, 7.2 mg), liraglutide (1.8, 3.0 mg), oral semaglutide (25 mg) and orforglipron (6, 12, 36 mg). The ATTAIN clinical trials evaluated orforglipron using capsule formulations; the FDA‐approved tablet formulation (Foundayo) has bioequivalent strengths of 5.5, 9, and 17.2 mg, respectively [9, 10]. Dual agonists (e.g., tirzepatide), crossover trials and post hoc or secondary analyses were excluded. Analyses used the efficacy estimand to reflect treatment effects under continued intervention exposure before treatment discontinuation or use of prohibited medications [11].

### 2.3 Outcomes of Interest

The primary outcome was the CEI, a ranking‐based composite measure (0–1) summarizing treatment performance across seven cardiometabolic endpoints: total body weight loss percentage (TBWL%), triglycerides (TG), HDL‐C, LDL‐C and absolute changes in waist circumference (WC), haemoglobin A1c (HbA1c) and systolic blood pressure (SBP). Higher CEI values indicate more favourable multidimensional cardiometabolic profiles.

Absolute body weight was not included because of its high correlation with TBWL% and WC. High‐sensitivity C‐reactive protein (hsCRP) and clinical cardiovascular or kidney outcomes were excluded due to inconsistent reporting, low‐risk population and limited follow‐up across obesity trials. The CEI integrates outcome‐specific rankings derived from NMA and should be interpreted as a descriptive summary of relative multidimensional performance rather than a calibrated measure of clinical benefit. Small numerical differences therefore reflect relative patterns rather than clinically meaningful superiority. Secondary outcomes included treatment effects for each individual CEI component.

### 2.4 Data Extraction

Two reviewers (J.C. and Y.G.) independently extracted data and assessed risk of bias via the Cochrane Risk of Bias tool, with disagreements resolved by a third reviewer (Y.L.).

### 2.5 Statistical Analysis

Given observed heterogeneity and outcome‐specific inconsistency within the network, random‐effects NMA models synthesized direct and indirect evidence, estimating placebo‐adjusted mean differences with 95% confidence intervals, presented in forest plots. Network inconsistency was assessed using node‐splitting and design‐by‐treatment models. Publication bias was evaluated with Egger's test. Lipid outcomes reported as treatment ratios or percent changes were log‐transformed for analysis and back‐transformed for interpretation. Subgroup analyses were performed by T2D status.

The CEI was constructed using surface under the cumulative ranking curve (SUCRA) values [12] derived from the NMA for seven cardiometabolic risk factors: TBWL%, WC, HbA1c, SBP, TG, HDL‐C and LDL‐C. SUCRA values represent the probability that a treatment ranks among the most effective for each outcome. These values were displayed in radar (origami) plots, with each axis representing one risk factor. The enclosed polygon area was normalized to a 0–1 scale to generate the CEI, with higher values indicating more favourable cardiometabolic profiles. Certainty of evidence was assessed using the GRADE framework (high, moderate, low or very low) [13]. Analyses used R (version 4.5.2, *netmeta* package) [14].

## 3 Results

### 3.1 Study Selection and Characteristics

Of 5022 citations, 19 RCTs (13 117 participants) met the inclusion criteria, enrolling adults with overweight or obesity, with or without T2D (Figure S1). Trials evaluated subcutaneous GLP‐1 receptor mono‐agonists (semaglutide 1.7, 2.4, 7.2 mg; liraglutide 1.8, 3.0 mg) and oral agents (oral semaglutide 25 mg; orforglipron 6, 12 and 36 mg). Baseline characteristics are summarized in Table S2. Mean age was 51.6 years, 61.6% female, BMI 31.6–40.5 kg/m^2^, and 33.0% had T2D. HbA1c ranged 5.5%–5.9% in non‐T2D and 6.7%–8.2% in T2D populations. Trial duration ranged from 44 to 104 weeks.

Between‐study heterogeneity was moderate to high overall and lower in T2D populations, except for HDL‐C (Table S3). Network inconsistency was observed for TBWL%, WC, HbA1c and LDL‐C (Table S4). Overall risk of bias was low (Table S5), and Egger's test did not suggest publication bias (Table S6).

### 3.2 Primary Outcome

**FIGURE 1:**
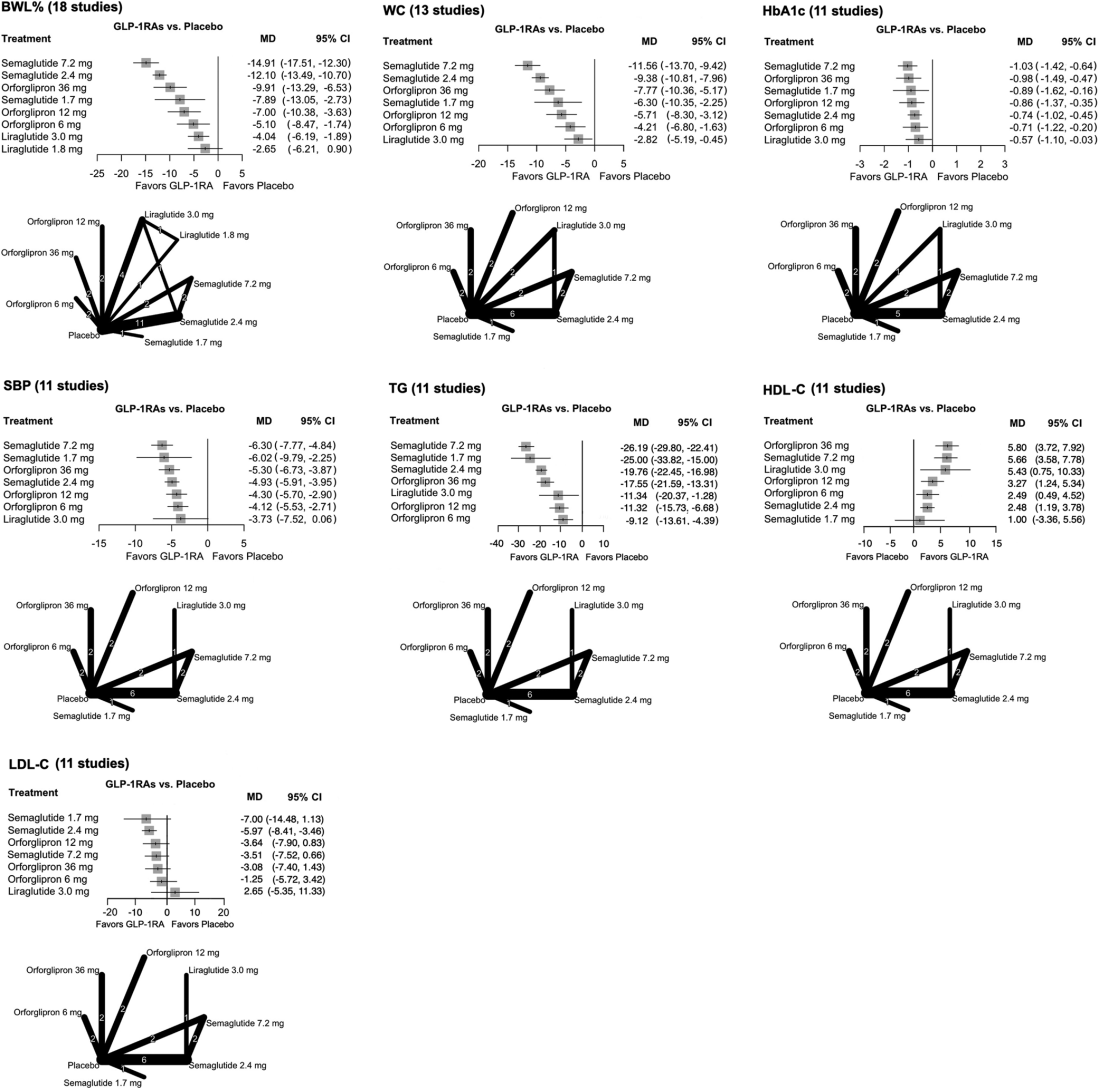
Forest and network plots of cardiometabolic profiles for glucagon‐like peptide‐1 (GLP‐1) receptor mono‐agonist versus placebo in adults with overweight or obesity. HbA1c, haemoglobin A1c; HDL‐C, high‐density lipoprotein cholesterol; LDL‐C, low‐density lipoprotein cholesterol; SBP, systolic blood pressure; TBWL%, total body weight loss percentage; TG, triglyceride; WC, waist circumference. *Top panels*: Forest plots showing mean differences (MD) and 95% confidence intervals (CI) for each treatment versus placebo across seven cardiometabolic risk factors (TBWL%, WC, HbA1c, SBP, TG, HDL‐C and LDL‐C). *Bottom panels*: Network plots visualizing the evidence structure. Nodes represent specific treatment arms and lines (edges) connecting nodes indicate direct head‐to‐head clinical trials, with line thickness proportional to the number of studies informing each comparison (thicker lines indicate more direct evidence). Closed loops show comparisons supported by both direct and indirect evidence, enabling statistical assessment of network consistency.

All treatments formed a coherent evidence network with placebo as the common comparator, with up to 18 RCTs contributing to individual endpoint analyses (Figure 1). Seven treatments with complete data across all endpoints were included in the origami plots: subcutaneous semaglutide 1.7, 2.4 and 7.2 mg; subcutaneous liraglutide 3.0 mg; and oral orforglipron 6, 12 and 36 mg.

CEI values (Table 1A, and Figure S2) indicated that higher‐dose GLP‐1 receptor mono‐agonists produced broader improvements across cardiometabolic domains. Semaglutide 7.2 mg had the highest CEI (0.86), followed by orforglipron 36 mg (0.68) and semaglutide 2.4 mg (0.66). Orforglipron 36 mg and semaglutide 2.4 mg showed comparable CEI values but differed in domain contributions: orforglipron showed stronger effects on glycemic control, HDL‐C and SBP, whereas semaglutide demonstrated larger effects on body weight, WC and LDL‐C. Dose–response patterns were evident for both semaglutide and orforglipron.

**TABLE 1:** Cardiometabolic efficacy index (CEI) for treatment evaluation in the network meta‐analysis (NMA).

A. Adults with overweight or obesity (with or without T2D)	
Treatment	CEI
Semaglutide 7.2 mg	0.86
Orforglipron 36 mg (17.2 mg tablet)	0.68
Semaglutide 2.4 mg	0.66
Semaglutide 1.7 mg	0.63
Orforglipron 12 mg (9 mg tablet)	0.49
Orforglipron 6 mg (5.5 mg tablet)	0.35
Liraglutide 3.0 mg	0.34
Placebo	0.04
B. Adults with overweight or obesity without T2D	
Treatment	CEI
Semaglutide 7.2 mg	0.88
Orforglipron 36 mg (17.2 mg tablet)	0.67
Semaglutide 2.4 mg	0.65
Oral semaglutide 25 mg	0.63
Orforglipron 12 mg (9 mg tablet)	0.50
Orforglipron 6 mg (5.5 mg tablet)	0.36
Liraglutide 3.0 mg	0.28
Placebo	0.02
C. Adults with overweight or obesity with T2D	
Treatment	CEI
Semaglutide 7.2 mg	0.85
Orforglipron 36 mg (17.2 mg tablet)	0.73
Semaglutide 2.4 mg	0.69
Orforglipron 12 mg (9 mg tablet)	0.47
Orforglipron 6 mg (5.5 mg tablet)	0.30
Placebo	0.10

*Note*: CEI is a ranking‐based composite derived from surface under the cumulative ranking curve value via random‐effects network meta‐analysis (NMA). For each treatment, SUCRA values for seven cardiometabolic risk factors (TBWL%, WC, HbA1c, SBP, TG, HDL‐C, and LDL‐C) are plotted on a radar chart and connected to form a polygon. The area of this polygon is calculated and normalized (0–1), representing the CEI value. Larger CEI values indicate greater overall cardiometabolic benefit across the analyzed endpoints, reflecting relative performance rather than absolute clinical effect.

Abbreviation: T2D, type 2 diabetes.

### 3.3 Secondary Outcomes

GLP‐1 receptor mono‐agonists consistently improved cardiometabolic endpoints versus placebo (Figure 1, Table S7). Semaglutide 7.2 mg achieved the largest reduction in TBWL% (MD −14.91%), followed by semaglutide 2.4 mg (−12.10%). Orforglipron showed a dose–response pattern (36 mg: −9.91%; 12 mg: −7.00%; 6 mg: −5.10%). WC reductions paralleled weight loss (semaglutide 7.2 mg: −11.56 cm; 2.4 mg: −9.38 cm; orforglipron 36 mg: −7.77 cm). HbA1c reductions were greatest with semaglutide 7.2 mg (−1.03%) and orforglipron 36 mg (−0.98%) across the overall population, regardless of T2D status. SBP reductions were also largest with semaglutide 7.2 mg (−6.30 mmHg), semaglutide 1.7 mg (−6.02 mmHg) and orforglipron 36 mg (−5.30 mmHg). Lipid outcomes improved modestly and dose‐dependently: TG decreased most with semaglutide 7.2 mg (−26.19%), HDL‐C increased modestly across therapies, with the largest increase observed with orforglipron 36 mg (5.8%) and LDL‐C reductions were largest with semaglutide 1.7 mg (−7.00%), with smaller or non‐significant effects for other agents. Certainty of evidence was rated as moderate to low across endpoints, generally higher for higher‐dose treatments (Table S8).

### 3.4 Subgroup Analysis

Across subgroups (non‐T2D versus T2D), CEI rankings were similar to the overall population, with semaglutide 7.2 mg ranking highest, followed by orforglipron 36 mg and semaglutide 2.4 mg (Table 1A–C, Figures S2–S6). Oral semaglutide 25 mg was included only in the non‐T2D subgroup, given the absence of T2D data; its CEI value (0.63) ranked fourth among all agents (Table 1B).

## 4 Discussion

This NMA provides an integrated assessment of cardiometabolic risk factors across oral and subcutaneous GLP‐1 receptor mono‐agonists in adults with overweight or obesity, with and without T2D. By synthesizing effects across multiple cardiometabolic domains, the analysis highlights patterns beyond weight reduction alone. Although several agents demonstrated improvements across multiple cardiometabolic domains, the magnitude and composition of treatment effects varied across drugs and dose regimens. Higher‐dose regimens (semaglutide 7.2 mg, orforglipron 36 mg and semaglutide 2.4 mg) demonstrated broader and more consistent improvements across cardiometabolic risk factors, whereas lower‐dose regimens showed more limited, domain‐specific effects. Treatments with similar overall profiles nevertheless differed in domain composition, reflecting trade‐offs between weight reduction, glycemic control, blood pressure and lipid modification. Because this analysis focused on GLP‐1 receptor mono‐agonists for mechanistic consistency, dual incretin agonists (e.g., tirzepatide) were excluded; future studies should compare multidimensional cardiometabolic profiles across all incretin‐based therapies.

This work extends prior meta‐analyses that primarily focused on weight reduction or individual outcomes. Earlier studies identified semaglutide as among the most effective agents for weight loss but did not systematically integrate cardiometabolic effects across risk factors or examine variation by formulation and dose [5, 15]. Using a unified efficacy estimand and the composite CEI, this analysis provides a framework for comparing multidimensional cardiometabolic profiles.

Observed differences between agents and doses likely reflect dose‐dependent pharmacologic effects on appetite, glycemia, blood pressure and lipid metabolism, as well as formulation‐related differences in exposure and tissue distribution [16, 17]. However, some differences may also reflect variability across trials rather than true drug‐specific effects. Subgroup analyses showed broadly similar CEI patterns across T2D status, although weight and lipid effects contributed more strongly in non‐T2D populations, whereas glycemic effects contributed more prominently in T2D populations.

The CEI requires cautious interpretation. As a ranking‐based composite derived from SUCRA values, it summarizes relative multidimensional performance rather than calibrated clinical effect sizes. Small numerical differences therefore reflect relative patterns rather than clinically meaningful superiority. Equal weighting of endpoints was adopted as a pragmatic approach in the absence of consensus on their relative prognostic importance. Importantly, CEI summarizes modification of cardiometabolic risk factors and does not imply differences in cardiovascular outcomes.

Several limitations should be considered. First, the analysis was restricted to GLP‐1 receptor mono‐agonists and excluded dual incretin agonists. Second, the study evaluated cardiometabolic risk factors rather than clinical outcomes such as cardiovascular or kidney events due to low‐risk populations and limited events reported in trials. Third, network inconsistency and substantial heterogeneity were observed for several outcomes, likely reflecting differences in baseline populations, treatment duration, drug regimens and clinical characteristics. Lifestyle co‐interventions and baseline comorbidity profiles also varied across trials and may have contributed to heterogeneity. Fourth, outcome reporting was inconsistent across studies, and some markers such as hsCRP were unavailable and therefore excluded from the CEI. Finally, the CEI focuses on efficacy‐related cardiometabolic risk factors and does not incorporate safety or tolerability outcomes.

In summary, GLP‐1 receptor mono‐agonists provide broad cardiometabolic benefits in adults with overweight or obesity, with effects varying across agents and doses. Higher‐dose regimens, including semaglutide 7.2 mg and orforglipron 36 mg (Foundayo 17.2 mg tablet), showed the most consistent multidimensional improvements.

## Funding

The study was supported in part by Eli Lilly and Company. The funder had no role in the study design, data collection, analysis and interpretation of the results.

## Conflicts of Interest

The authors declare no conflicts of interest.

## Supporting information


**Table S1:** Search strategy.
**Table S2:** Baseline characteristics of 19 randomized placebo‐controlled glucagon‐like peptide‐1 (GLP‐1) receptor mono‐agonist trials in adults with overweight or obesity.
**Table S3:** Between‐study heterogeneity for seven cardiometabolic risk factors in the network meta‐analysis.
**Table S4:** Network inconsistency for seven cardiometabolic risk factors in the network meta‐analysis (NMA) using the design‐by‐treatment interaction model.
**Table S5:** Risk of bias evaluation of randomized controlled trials (RCTs) included in the network meta‐analysis (NMA).
**Table S6:** *p* value from Egger's test for seven cardiometabolic risk factors in the network meta‐analysis (NMA).
**Table S7:** Cardiometabolic efficacy index (CEI) for treatment evaluation in the network meta‐analysis (NMA).
**Table S8:** League table of direct and indirect comparisons among placebo and active treatments for adults with overweight or obesity in the network meta‐analysis (NMA).
**Table S9:** GRADE checklist.
**Figure S1:** PRISMA flow diagram displaying results of the literature search.
**Figure S2:** Pairwise‐origami plot summarizing multidimensional efficacy across seven cardiometabolic risk factors in adults with overweight or obesity.
**Figure S3:** Forest and network plots of cardiometabolic risk factors for glucagon‐Like peptide‐1 (GLP‐1) receptor mono‐agonist versus placebo in adults with overweight or obesity without T2D.
**Figure S4:** Pairwise‐origami plot summarizing multidimensional efficacy across seven cardiometabolic risk factors in adults with overweight or obesity without T2D.
**Figure S5:** Forest and network plots of cardiometabolic risk factors for glucagon‐Like peptide‐1 (GLP‐1) receptor mono‐agonist versus placebo in adults with overweight or obesity with T2D.
**Figure S6:** Pairwise‐origami plot summarizing multidimensional efficacy across seven cardiometabolic risk factors in adults with overweight or obesity with T2D.

## Data Availability

Data are derived from public domain resources.
