# Supplementary material for: Cardiometabolic Profiles of Oral and Subcutaneous Glucagon‐Like Peptide‐1 Receptor Mono‐Agonists in Adults With Overweight or Obesity: A Systematic Review and Network Meta‐Analysis
Source: Diabetes Obes Metab. 2026 Apr 16;28(7):5761–6. doi: 10.1111/dom.70742 (PMC13243969; doi:10.1111/dom.70742)
Supplement: Supplementary file 1 — Table S1: Search strategy. Table S2: Baseline characteristics of 19 randomized placebo‐controlled glucagon‐like peptide‐1 (GLP‐1) receptor mono‐agonist trials in adults with overweight or obesity. Table S3: Between‐study heterogeneity for seven cardiometabolic risk factors in the network meta‐analysis. Table S4: Network inconsistency for seven cardiometabolic risk factors in the network meta‐analysis (NMA) using the design‐by‐treatment interaction model. Table S5: Risk of bias evaluation of randomized controlled trials (RCTs) included in the network meta‐analysis (NMA). Table S6: p value from Egger's test for seven cardiometabolic risk factors in the network meta‐analysis (NMA). Table S7: Cardiometabolic efficacy index (CEI) for treatment evaluation in the network meta‐analysis (NMA). Table S8: League table of direct and indirect comparisons among placebo and active treatments for adults with overweight or obesity in the network meta‐analysis (NMA). Table S9: GRADE checklist. Figure S1: PRISMA flow diagram displaying results of the literature search. Figure S2: Pairwise‐origami plot summarizing multidimensional efficacy across seven cardiometabolic risk factors in adults with overweight or obesity. Figure S3: Forest and network plots of cardiometabolic risk factors for glucagon‐Like peptide‐1 (GLP‐1) receptor mono‐agonist versus placebo in adults with overweight or obesity without T2D. Figure S4: Pairwise‐origami plot summarizing multidimensional efficacy across seven cardiometabolic risk factors in adults with overweight or obesity without T2D. Figure S5: Forest and network plots of cardiometabolic risk factors for glucagon‐Like peptide‐1 (GLP‐1) receptor mono‐agonist versus placebo in adults with overweight or obesity with T2D. Figure S6: Pairwise‐origami plot summarizing multidimensional efficacy across seven cardiometabolic risk factors in adults with overweight or obesity with T2D. [file DOM-28-5761-s001.docx]

**Supplementary materials**

**Cardiometabolic Profiles of Oral and Subcutaneous Glucagon-like Peptide-1 Receptor Mono-agonists in Adults with Overweight or Obesity: A Systematic Review and Network Meta-Analysis**

**Table S1.** Search strategy

**Table S2.** Baseline characteristics of 19 randomized placebo-controlled glucagon-like peptide-1 (GLP-1) receptor mono-agonist trials in adults with overweight or obesity

**Table S3.** Between-study heterogeneity for seven cardiometabolic risk factors in the network meta‑analysis

**Table S4.** Network inconsistency for seven cardiometabolic risk factors in the network meta‑analysis (NMA) using the design-by-treatment interaction model

**Table S5.** Risk of bias evaluation of randomized controlled trials (RCTs) included in the network meta-analysis (NMA)

**Table S6.** P value from Egger’s test for seven cardiometabolic risk factors in the network meta‑analysis (NMA)

**Table S7.** League table of direct and indirect comparisons among placebo and active treatments for adults with overweight or obesity in the network meta-analysis (NMA)

**Table S8.** GRADE checklist

**Figure S1.** PRISMA flow diagram displaying results of the literature search

**Figure S2.** Pairwise-origami plot summarizing multidimensional efficacy across seven cardiometabolic risk factors in adults with overweight or obesity

**Figure S3.** Forest and network plots of cardiometabolic risk factors for glucagon-Like peptide-1 (GLP-1) receptor mono-agonist versus placebo in adults with overweight or obesity without T2D

**Figure S4.** Pairwise-origami plot summarizing multidimensional efficacy across seven cardiometabolic risk factors in adults with overweight or obesity without T2D

**Figure S5.** Forest and network plots of cardiometabolic risk factors for glucagon-Like peptide-1 (GLP-1) receptor mono-agonist versus placebo in adults with overweight or obesity with T2D

**Figure S6.** Pairwise-origami plot summarizing multidimensional efficacy across seven cardiometabolic risk factors in adults with overweight or obesity with T2D

**Table S1.** Search strategy

| PUBMED QUERY (n = 738) | |
| --- | --- |
| #1 | "orforglipron"[Title/Abstract] OR "LY3502970"[Title/Abstract] OR "liraglutide"[MeSH Terms] OR "liraglutide"[Title/Abstract] OR "semaglutide"[Title/Abstract] |
| #2 | "randomized controlled trial"[Publication Type] OR "controlled clinical trial"[Publication Type] OR "Randomized Controlled Trials as Topic"[MeSH] OR "randomized trial"[Title/Abstract] OR “randomization”[tiab] OR “randomized”[tiab] OR “randomised”[tiab] OR "RCT"[Title/Abstract] |
| #3 | #1 and #2 |
| #4 | "obesity"[MeSH Terms] OR "obesity"[Title/Abstract] OR “obese”[Title/Abstract] |
| #5 | "overweight"[MeSH Terms] OR "overweight"[Title/Abstract] OR "over weight"[Title/Abstract] |
| #6 | type 2 diabetes'/exp OR 'type 2 diabetes':ti,ab,kw OR 'T2D'/exp OR 'T2D':ti,ab,kw OR 'diabetes mellitus, type 2'/exp OR 'diabetes mellitus, type 2':ti,ab,kw OR 'non insulin dependent diabetes mellitus'/exp |
| #7 | #4 OR #5 OR #6 |
| #8 | #3 and #7 |
| Filters | Clinical trial; Randomized Controlled Trial; from 2014 – 2025; English |
| EMBASE QUERY (n= 841) | |
| #1 | 'orforglipron'/exp OR 'orforglipron':ti,ab,kw OR 'ly3502970':ti,ab,kw OR 'liraglutide'/exp OR 'liraglutide':ti,ab,kw OR 'semaglutide'/exp OR 'semaglutide':ti,ab,kw |
| #2 | 'randomized controlled trial'/exp OR 'rct':ti,ab,kw OR 'RCT':ti,ab,kw OR 'randomization':ti,ab,kw OR 'randomized':ti,ab,kw OR 'randomised':ti,ab,kw |
| #3 | #1 and #2 |
| #4 | 'obesity'/exp OR 'obesity':ti,ab,kw |
| #5 | 'overweight'/exp OR 'overweight':ti,ab,kw OR 'over weight':ti,ab,kw |
| #6 | 'type 2 diabetes'/exp OR 'type 2 diabetes':ti,ab,kw OR 'T2D'/exp OR 'T2D':ti,ab,kw OR 'diabetes mellitus, type 2'/exp OR 'diabetes mellitus, type 2':ti,ab,kw OR 'non insulin dependent diabetes mellitus'/exp |
| #7 | #4 OR #5 OR #6 |
| #8 | #3 AND #7 |
| Filters | 'human'/de; 'randomized controlled trial'/de; 'clinical trial'/de; (2014:py OR 2015:py OR 2016:py OR 2017:py OR 2018:py OR 2019:py OR 2020:py OR 2021:py OR 2022:py OR 2023:py OR 2024:py OR 2025:py) |
| CENTRAL QUERY (n= 3,443) | |
| #1 | (orforglipron OR LY3502970):ti,ab,kw (Word variations have been searched) |
| #2 | (semaglutide):ti,ab,kw (Word variations have been searched) |
| #3 | (liraglutide):ti,ab,kw (Word variations have been searched) |
| #4 | MeSH descriptor: [Liraglutide] explode all trees |
| #5 | #1 OR #2 OR #3 OR #4 |
| #6 | MeSH descriptor: [Randomized Controlled Trial] explode all trees |
| #7 | (randomized controlled trial OR RCT OR randomisation OR randomized OR randomised):ti,ab,kw (Word variations have been searched) |
| #8 | #6 OR #7 |
| #9 | #5 AND #8 |
| #10 | (obesity OR obese):ti,ab,kw (Word variations have been searched) |
| #11 | MeSH descriptor: [Obesity] explode all trees |
| #12 | #10 OR #11 |
| #13 | (overweight or "over weight"):ti,ab,kw (Word variations have been searched) |
| #14 | MeSH descriptor: [Overweight] explode all trees |
| #15 | #13 OR #14 |
| #16 | ("diabetes mellitus type 2" OR "type 2 diabetes" OR "T2D"):ti,ab,kw (Word variations have been searched) |
| #17 | MeSH descriptor: [Diabetes Mellitus, Type 2] explode all trees |
| #18 | #16 OR #17 |
| #19 | #12 OR #15 OR #18 |
| #20 | #9 AND #19 |
| Filters | with Publication Year from 2014 to 2025, in Trials (Word variations have been searched) |

**Table S2.** Baseline characteristics of 19 randomized placebo-controlled glucagon-like peptide-1 (GLP-1) receptor mono-agonist trials in adults with overweight or obesity

| Source | | Treatment | | N | Dur  (Wk) | | | Age  (yr) | | White  (%) | Female  (%) | | | Body  weight  (kg) | | BMI  (kg/m^2^) | | WC  (cm) | | HbA1c (%) | | T2D  (%) | | | HT  (%) | DLP  (%) | |  |
| --- | --- | --- | --- | --- | --- | --- | --- | --- | --- | --- | --- | --- | --- | --- | --- | --- | --- | --- | --- | --- | --- | --- | --- | --- | --- | --- | --- | --- |
| Wharton 2025^1^  NCT05869903 | Orforglipron 6 mg | | 559 | | | 72 | 44.9 (12.1) | | 57.1 | | | 64.9 | 103.2 (21.7) | | 37.0 (6.5) | | 112.2 (14.1) | | 5.6 (0.4) | | 0.0 | |  | 40.7 | | 38.5 |  |  |
|  | Orforglipron 12 mg | | 559 | | |  | 45.4 (12.6) | | 56.6 | | | 64.4 | 102.2 (21.6) | | 36.7 (6.5) | | 112.0 (14.2) | | 5.6 (0.3) | | 0.0 | |  | 38.1 | | 38.9 |  |  |
|  | Orforglipron 36 mg | | 549 | | |  | 44.9 (11.9) | | 54.4 | | | 63.7 | 103.1 (23.2) | | 36.9 (6.7) | | 112.4 (15.3) | | 5.6 (0.3) | | 0.0 | |  | 39.5 | | 38.9 |  |  |
|  | Placebo | | 654 | | |  | 45.1 (11.9) | | 57.5 | | | 64.1 | 103.9 (22.0) | | 37.1 (6.3) | | 112.8 (14.5) | | 5.6 (0.3) | | 0.0 | |  | 39.8 | | 40.7 |  |  |
| Horn 2025^2^  NCT05872620 | Orforglipron 6 mg | | 329 | | | 72 | 56.8 (10.4) | | 72.3 | | | 45.6 | 102.3 (22.7) | | 35.9 (7.0) | | 116.8 (15.1) | | 8.0 (0.7) | | 100.0 | |  | 76.6 | | 70.5 |  |  |
|  | Orforglipron 12 mg | | 332 | | |  | 56.2 (10.5) | | 70.8 | | | 46.7 | 102.7 (21.3) | | 36.1 (6.3) | | 116.2 (13.4) | | 8.1 (0.8) | | 100.0 | |  | 70.2 | | 72.9 |  |  |
|  | Orforglipron 36 mg | | 322 | | |  | 58.1 (10.8) | | 70.8 | | | 47.8 | 99.8 (23.0) | | 35.1 (6.5) | | 114.7 (15.1) | | 8.15 (0.7) | | 100.0 | |  | 76.4 | | 69.9 |  |  |
|  | Placebo | | 630 | | |  | 56.5 (10.9) | | 70.2 | | | 47.3 | 101.2 (22.6) | | 35.5 (6.5) | | 115.0 (14.6) | | 8.0 (0.8) | | 100.0 | |  | 74.6 | | 70.0 |  |  |
| Davies 2015^3^  NCT01272232 | Liraglutide 3.0 mg | | 423 | | | 56 | 55.0 (10.8) | | 83.5 | | | 48.0 | 105.7 (21.9) | | 37.1 (6.5) | | 118.0 (14.4) | | 7.9 (0.8) | | 100.0 | |  | NA | | NA |  |  |
|  | Liraglutide 1.8 mg | | 211 | | |  | 54.9 (10.7) | | 83.9 | | | 48.8 | 105.8 (21.0) | | 37.0 (6.9) | | 117.5 (14.7) | | 8.0 (0.8) | | 100.0 | |  | NA | | NA |  |  |
|  | Placebo | | 212 | | |  | 54.7  (9.8) | | 82.5 | | | 54.2 | 106.5 (21.3) | | 37.4 (7.1) | | 117.3 (14.0) | | 7.9 (0.8) | | 100.0 | |  | NA | | NA |  |  |
| Wadden 2020^4^  NCT02963935 | Liraglutide 3.0 mg | | 114 | | | 56 | 45.4 (11.6) | | 78.9 | | | 83.8 | 108.5 (22.1) | | 39.3 (6.8) | | 116.0 (14.4) | | 5.5 (0.4) | | 0.0 | |  | NA | | NA |  |  |
|  | Placebo | | 103 | | |  | 49.0 (11.2) | | 82.1 | | | 82.9 | 106.7 (22.0) | | 38.7 (7.2) | | 115.0 (15.6) | | 5.5 (0.4) | | 0.0 | |  | NA | | NA |  |  |
| Garvey 2020^5^  NCT02963922 | Liraglutide 3.0 mg | | 198 | | | 56 | 55.9 (11.3) | | 87.9 | | | 54.5 | 100.6 (20.8) | | 35.9 (6.5) | | 114.8 (13.7) | | 7.9 (1.1) | | 100.0 | |  | NA | | NA |  |  |
|  | Placebo | | 198 | | |  | 57.6 (10.4) | | 90.9 | | | 50.0 | 98.9 (19.9) | | 35.3 (5.8) | | 114.2 (13.2) | | 8.0 (1.0) | | 100.0 | |  | NA | | NA |  |  |
| Wilding 2021^6^  NCT03548935 | Semaglutide 2.4 mg | | 1306 | | | 68 | 46.0 (13.0) | | 74.5 | | | 73.1 | 105.4 (22.1) | | 37.8 (6.7) | | 114.6 (14.8) | | 5.7 (0.3) | | 0.0 | |  | 36.1 | | 38.2 |  |  |
|  | Placebo | | 655 | | |  | 47.0 (12.0) | | 76.2 | | | 76.0 | 105.2 (21.5) | | 38.0 (6.5) | | 114.8 (14.4) | | 5.7 (0.3) | | 0.0 | |  | 35.7 | | 34.5 |  |  |
| Davies 2021^7^  NCT03552757 | Semaglutide 2.4 mg | | 351 | | | 68 | 55.0 (11.0) | | 58.7 | | | 55.2 | 99.9 (22.5) | | 35.9 (6.4) | | 114.5 (14.3) | | 8.1 (0.8) | | 100.0 | |  | NA | | NA |  |  |
|  | Placebo | | 340 | | |  | 55.0 (11.0) | | 60.0 | | | 47.1 | 100.5 (20.9) | | 35.9 (6.5) | | 115.5 (13.9) | | 8.1 (0.8) | | 100.0 | |  | NA | | NA |  |  |
| Wadden 2021^8^  NCT03611582 | Semaglutide 2.4 mg | | 339 | | | 68 | 46.0 (13.0) | | 75.4 | | | 77.4 | 106.9 (22.8) | | 38.1 (6.7) | | 113.6 (15.1) | | NA | | 0.0 | |  | 35.6 | | 35.6 |  |  |
|  | Placebo | | 166 | | |  | 46.0 (13.0) | | 77.5 | | | 88.2 | 103.7 (22.9) | | 37.8 (6.9) | | 111.8 (16.2) | | NA | | 0.0 | |  | 32.8 | | 32.8 |  |  |
| Garvey 2022^9^  NCT03693430 | Semaglutide 2.4 mg | | 132 | | | 104 | 47.3 (11.7) | | 92.8 | | | 80.9 | 105.6 (20.8) | | 38.6 (6.7) | | 115.8 (14.3) | | 5.7 (0.3) | | 0.0 | |  | 36.8 | | 38.2 |  |  |
|  | Placebo | | 109 | | |  | 47.4 (10.3) | | 93.4 | | | 74.3 | 106.5 (23.1) | | 38.5 (7.2) | | 115.7 (15.5) | | 5.7 (0.4) | | 0.0 | |  | 40.8 | | 32.2 |  |  |
| Kadowaki 2022^10^  NCT03811574 | Semaglutide 2.4 mg | | 186 | | | 68 | 52.0 (12.0) | | NA | | | 37.0 | 86.9 (16.5) | | 32.0 (4.6) | | 103.8 (11.8) | | 6.4 (1.2) | | 25.0 | |  | 76.0 | | 90.0 |  |  |
|  | Semaglutide 1.7 mg | | 93 | | |  | 51.0 (10.0) | | NA | | | 37.0 | 86.1 (11.9) | | 31.6 (3.7) | | 101.4 (8.8) | | 6.4 (1.1) | | 25.0 | |  | 73.0 | | 87.0 |  |  |
|  | Placebo | | 98 | | |  | 50.0 (9.0) | | NA | | | 26.0 | 90.2 (15.1) | | 31.9 (4.2) | | 103.8 (9.9) | | 6.4 (1.1) | | 25.0 | |  | 72.0 | | 79.0 |  |  |
| Mu 2024^11^  NCT04251156 | Semaglutide 2.4 mg | | 204 | | | 44 | 41.0 (11.0) | | 9.0 | | | 45.0 | 96.4 (17.9) | | 34.0 (4.9) | | 108.5 (11.8) | | 6.2 (1.1) | | 26.0 | |  | 45.0 | | 61.0 |  |  |
|  | Placebo | | 98 | | |  | 40.0 (11.0) | | 7.0 | | | 47.0 | 96.2 (17.3) | | 34.0 (4.6) | | 107.0 (10.6) | | 6.3 (1.2) | | 25.0 | |  | 48.0 | | 60.0 |  |  |
| Rubino 2022^12^ NCT04074161 | Semaglutide 2.4 mg | | 95 | | | 68 | 48.0 (14.0) | | 74.6 | | | 81.0 | 102.5 (25.3) | | 37.0 (7.4) | | 111.8 (16.3) | | 5.5 (0.3) | | 0.0 | |  | 38.1 | | 47.6 |  |  |
|  | Liraglutide 3.0 mg | | 90 | | |  | 49.0 (13.0) | | 74.8 | | | 76.4 | 103.7 (22.5) | | 37.2 (6.4) | | 113.5 (15.0) | | 5.5 (0.3) | | 0.0 | |  | 43.3 | | 51.2 |  |  |
|  | Placebo | | 66 | | |  | 51.0 (12.0) | | 70.6 | | | 77.6 | 108.8 (23.1) | | 38.8 (6.5) | | 115.4 (15.1) | | 5.6 (0.4) | | 0.0 | |  | 45.9 | | 42.4 |  |  |
| Bliddal 2024^13^  NCT05064735 | Semaglutide 2.4 mg | | 271 | | | 68 | 56.0 (10.0) | | 62.0 | | | 84.1 | 108.7 (24.1) | | 40.5 (7.3) | | 118.3 (15.8) | | NA | | 0.0 | |  | 47.2 | | 29.5 |  |  |
|  | Placebo | | 136 | | |  | 56.0 (10.0) | | 58.8 | | | 76.5 | 108.5 (24.5) | | 40.0 (7.1) | | 119.7 (15.9) | | NA | | 0.0 | |  | 50.0 | | 32.4 |  |  |
| McGowan 2024^14^  NCT05040971 | Semaglutide 2.4 mg | | 138 | | | 52 | 53.0 (11.0) | | 90.0 | | | 72.0 | 111.9 (21.5) | | 39.9 (6.6) | | 120.1 (14.8) | | 5.9 (0.3) | | 0.0 | |  | 46.0 | | 46.0 |  |  |
|  | Placebo | | 69 | | |  | 53.0 (11.0) | | 86.0 | | | 68.0 | 111 (23.5) | | 40.4 (7.6) | | 119.9 (14.7) | | 5.9 (0.3) | | 0.0 | |  | 46.0 | | 32.0 |  |  |
| Wharton 2025^15^  NCT05646706 | Semaglutide 7.2 mg | | 739 | | | 72 | 47.0 (12.0) | | 85.5 | | | 74.9 | 112.4 (23.8) | | 39.8 (7.0) | | 118.4 (15.8) | | 5.7(0.3) | | 0.0 | |  | 42.9 | | 27.6 |  |  |
|  | Semaglutide 2.4 mg | | 149 | | |  | 46.0 (12.0) | | 87.6 | | | 68.2 | 116.5 (26.2) | | 40.5 (7.8) | | 120.3 (16.9) | | 5.6 (0.4) | | 0.0 | |  | 37.3 | | 27.4 |  |  |
|  | Placebo | | 128 | | |  | 48.0 (12.0) | | 83.6 | | | 73.1 | 112.4 (22.8) | | 39.7 (6.6) | | 118.6 (14.5) | | 5.7(0.3) | | 0.0 | |  | 39.3 | | 29.9 |  |  |
| Lingvay 2025^16^  NCT05649137 | Semaglutide 7.2 mg | | 235 | | | 72 | 57.0 (10.0) | | 84.0 | | | 54.1 | 110.5 (22.9) | | 38.7 (7.1) | | 121.8 (15.2) | | 8.0 (0.8) | | 100.0 | |  | 79.8 | | 70.0 |  |  |
|  | Semaglutide | | 82 | | |  | 58.0 (10.0) | | 80.6 | | | 45.6 | 107.0 (23.0) | | 37.7 (6.4) | | 119.1 (13.2) | | 8.1 (0.9) | | 100.0 | |  | 81.6 | | 64.1 |  |  |
|  | Placebo | | 77 | | |  | 55.0 (10.0) | | 85.3 | | | 51.0 | 112.1 (22.9) | | 39.0 (7.6) | | 123.9 (15.5) | | 8.2 (0.9) | | 100.0 | |  | 77.5 | | 62.7 |  |  |
| Kosiborod 2023^17^  NCT04788511 | Semaglutide 2.4 mg | | 263 | | | 52 | 70.0  (62.0-75.0) | | 97.0 | | | 56.7 | 104.7  (92.4-120.1) | | 37.2  (33.9-41.1) | | 119.0  (110.5-127.1) | | NA | | 0.0 | |  | 82.1 | | NA |  |  |
|  | Placebo | | 266 | | |  | 69.0  (62.0-75.0) | | 94.7 | | | 55.6 | 105.3  (92.4-122) | | 36.9  (33.3-41.6) | | 120.0  (110.5-129) | | NA | | 0.0 | |  | 81.6 | | NA |  |  |
| Kosiborod 2024^18^  NCT04916470 | Semaglutide 2.4 mg | | 310 | | | 52 | 69.0  (62.0-74.0) | | 81.0 | | | 41.3 | 103.8  (91.9-119.0) | | 36.9  (33.6-41.5) | | 122.0  (113.0-131.5) | | 6.7  (6.2-7.4) | | 100.0 | |  | 82.3 | | NA |  |  |
|  | Placebo | | 306 | | |  | 70.0  (63.0-75.0) | | 87.6 | | | 47.4 | 101.7  (89.5-116.4) | | 36.9  (33.5-41.1) | | 118.5  (111.0-129.0) | | 6.9  (6.2-7.7) | | 100.0 | |  | 88.6 | | NA |  |  |
| Wharton 2025^19^  NCT05564117 | Oral semaglutide 25 mg | | 157 | | | 64 | 48.0 (13.0) | | 92.7 | | | 75.6 | 106.4 (23.5) | | 37.5 (6.7) | | 114.0 (15.8) | | 5.7 (0.4) | | 0.0 | |  | 44.4 | | 31.2 |  |  |
|  | Placebo | | 70 | | |  | 47.0 (13.0) | | 89.2 | | | 85.3 | 104.8 (19.7) | | 37.8 (6.1) | | 113.6 (14.7) | | 5.7 (0.3) | | 0.0 | |  | 41.2 | | 28.4 |  |  |

**Note:** Values are reported as mean (SD) or range for Age, BMI, HbA1c, body weight and WC.

**Abbreviations:** N, number of patients; Dur, duration of treatment; wk, week; yr, year BMI, body mass index; WC, waist circumference; HbA1c, haemoglobin A1c; T2D, type 2 diabetes; HT, hypertension; DLP, Dyslipidemia.

# **Table S3.** Between-study heterogeneity for seven cardiometabolic risk factors in the network meta‑analysis

| **Outcome** | **No. of studies** | **tau^2** | **tau** | **I^2 (95% CI)** | **Heterogeneity category** |
| --- | --- | --- | --- | --- | --- |
| **Adults with obesity or overweight** | | | | | |
| TBWL% | 18 | 5.733 | 2.394 | 93.2% [91.0%; 94.9%] | Very High |
| WC | 13 | 3.301 | 1.817 | 88.9% [83.2%; 92.7%] | Very High |
| HbA1c | 11 | 0.132 | 0.363 | 98.9% [98.6%; 99.1%] | Very High |
| SBP | 11 | 0.479 | 0.692 | 32.8% [0.0%; 66.1%] | Moderate |
| TG | 11 | 0.001 | 0.027 | 42.5% [0.0%; 70.8%] | Moderate |
| HDL-C | 11 | <0.001 | 0.010 | 38.4% [0.0%; 68.9%] | Moderate |
| LDL-C | 11 | 0.001 | 0.027 | 61% [26.8%; 79.2%] | High |
| **Adults with obesity or overweight without T2D** | | | | | |
| TBWL% | 12 | 0.647 | 0.804 | 48% [0.0%; 74.9%] | Moderate |
| WC | 9 | <0.001 | <0.001 | 0.0% [0.0%; 79.2%]† | Low |
| HbA1c | 8 | 0.001 | 0.038 | 72.9% [23.5%; 90.4%] | High |
| SBP | 8 | 1.014 | 1.007 | 46.8% [0.0%; 82.3%] | Moderate |
| TG | 8 | 0.001 | 0.021 | 30.7% [0.0%; 75.0%] | Moderate |
| HDL-C | 8 | <0.001 | 0.006 | 16.3% [0.0%; 87.2%] | Low |
| LDL-C | 8 | <0.001 | <0.001 | 0% [0.0%; 84.7%]† | Low |
| **Adults with obesity or overweight with T2D** | | | | | |
| TBWL% | 6 | <0.001 | <0.001 | 0.0% [0.0%; 84.7%] | Low |
| WC | 4 | <0.001 | <0.001 | 0.0% | Low |
| HbA1c | 4 | 0.001 | 0.027 | 5.6% | Low |
| SBP | 3 | <0.001 | <0.001 | 0.0% | Low |
| TG | 3 | <0.001 | <0.001 | 0.0% | Low |
| HDL-C | 3 | 0.001 | 0.035 | 79.4% [10.9%; 95.2%] | Very High |
| LDL-C | 3 | <0.001 | <0.001 | 0.0% | Low |
| **Abbreviation:** CI, confidence interval; TBWL%, total body weight loss percentage; WC, waist circumference; HbA1c, haemoglobin A1c; SBP, systolic blood pressure; TG, triglyceride; HDL-C, high-density lipoprotein cholesterol; LDL-C, low-density lipoprotein cholesterol; T2D, type 2 diabetes.  **Note:** Categories based on prespecified thresholds: low (I² <25%), moderate (25–50%), high (50–75%), very high (>75%). † I² = 0% with very wide confidence intervals indicates limited power to detect between‑study heterogeneity rather than definitive homogeneity. | | | | | |

# **Table S4.** Network inconsistency for seven cardiometabolic risk factors in the network meta‑analysis (NMA) using the design-by-treatment interaction model

# Adults with obesity or overweight

| **Component** | **Q statistic** | **Degrees of freedom** | **P value** |
| --- | --- | --- | --- |
| **TBWL%** | | | |
| Total | 309.6 | 21 | < 0.001 |
| Within-design | 301.6 | 18 | < 0.001 |
| Between-design | 7.9 | 3 | 0.048 |
| **WC** | | | |
| Total | 117.4 | 13 | < 0.001 |
| Within designs | 114.6 | 11 | < 0.001 |
| Between designs | 2.8 | 2 | 0.247 |
| **HbA1c** | | | |
| Total | 987.3 | 11 | < 0.001 |
| Within designs | 969.5 | 9 | < 0.001 |
| Between designs | 17.8 | 2 | 0.001 |
| **SBP** | | | |
| Total | 16.4 | 11 | 0.128 |
| Within designs | 16.2 | 10 | 0.094 |
| Between designs | 0.1 | 1 | 0.711 |
| **TG** | | | |
| Total | 19.1 | 11 | 0.059 |
| Within designs | 17.3 | 10 | 0.067 |
| Between designs | 1.8 | 1 | 0.180 |
| **HDL-C** | | | |
| Total | 17.9 | 11 | 0.085 |
| Within designs | 17.7 | 10 | 0.060 |
| Between designs | 0.2 | 1 | 0.689 |
| **LDL-C** | | | |
| Total | 28.2 | 11 | 0.003 |
| Within designs | 28.2 | 10 | 0.002 |
| Between designs | 0.0 | 1 | 0.906 |

#

1. Adults with obesity or overweight without T2D

| **Component** | **Q statistic** | **Degrees of freedom** | **P value** |
| --- | --- | --- | --- |
| **TBWL%** | | | |
| Total | 17.3 | 9 | 0.044 |
| Within designs | 15.0 | 7 | 0.036 |
| Between designs | 2.3 | 2 | 0.319 |
| **WC** | | | |
| Total | 1.8 | 4 | 0.782 |
| Within designs | 1.6 | 2 | 0.459 |
| Between designs | 0.2 | 2 | 0.908 |
| **HbA1c** | | | |
| Total | 11.1 | 3 | 0.011 |
| Within designs | 8.8 | 2 | 0.012 |
| Between designs | 2.2 | 1 | 0.136 |
| **SBP** | | | |
| Total | 5.6 | 3 | 0.131 |
| Within designs | 5.6 | 2 | 0.060 |
| Between designs | 0.0 | 1 | 0.939 |
| **TG** | | | |
| Total | 4.3 | 3 | 0.228 |
| Within designs | 4.0 | 2 | 0.138 |
| Between designs | 0.4 | 1 | 0.543 |
| **HDL-C** | | | |
| Total | 3.6 | 3 | 0.310 |
| Within designs | 3.1 | 2 | 0.218 |
| Between designs | 0.5 | 1 | 0.465 |
| **LDL-C** | | | |
| Total | 2.3 | 3 | 0.521 |
| Within designs | 2.3 | 2 | 0.324 |
| Between designs | 0.0 | 1 | 0.931 |

1. Adults with obesity or overweight with T2D

| **Component** | **Q statistic** | **Degrees of freedom** | **P value** |
| --- | --- | --- | --- |
| **TBWL%** | | | |
| Total | 2.9 | 4 | 0.579 |
| Within designs | 2.4 | 2 | 0.296 |
| Between designs | 0.4 | 2 | 0.802 |
| **WC** | | | |
| Total | 0.8 | 1 | 0.368 |
| Within designs | 0.0 | 0 | -- |
| Between designs | 0.8 | 1 | 0.368 |
| **HbA1c** | | | |
| Total | 1.1 | 1 | 0.303 |
| Within designs | 0.0 | 0 | -- |
| Between designs | 1.1 | 1 | 0.303 |
| **SBP** | | | |
| Total | 0.3 | 1 | 0.600 |
| Within designs | 0.0 | 0 | -- |
| Between designs | 0.3 | 1 | 0.600 |
| **TG** | | | |
| Total | 0.3 | 1 | 0.566 |
| Within designs | 0.0 | 0 | -- |
| Between designs | 0.3 | 1 | 0.566 |
| **HDL-C** | | | |
| Total | 4.9 | 1 | 0.028 |
| Within designs | 0.0 | 0 | -- |
| Between designs | 4.9 | 1 | 0.028 |
| **LDL-C** | | | |
| Total | 0.0 | 1 | 0.900 |
| Within designs | 0.0 | 0 | -- |
| Between designs | 0.0 | 1 | 0.900 |

**Abbreviation:** TBWL%, total body weight loss percentage; WC, waist circumference; HbA1c, haemoglobin A1c; SBP, systolic blood pressure; TG, triglyceride; HDL-C, high-density lipoprotein cholesterol; LDL-C, low-density lipoprotein cholesterol; T2D, type 2 diabetes.

# **Table S5.** Risk of bias evaluation of randomized controlled trials (RCTs) included in the network meta-analysis (NMA)

|  | **Sequence generation** | **Allocation concealment** | **Blinding participants** | **Blinding of outcome assessors** | **Incomplete outcome data** | **Selective outcome reporting** | **Other bias** | **Overall risk of bias** |
| --- | --- | --- | --- | --- | --- | --- | --- | --- |
| **Wharton 2025^a^** | Low | Unclear | Low | Low | Low | Low | Unclear | Low |
| **Horn 2025** | Low | Low | Low | Low | Low | Low | Unclear | Low |
| **Davies 2015** | Low | Low | Low | Low | Low | Low | Unclear | Low |
| **Wadden 2020** | Low | Low | Low | Low | Low | Low | Unclear | Low |
| **Garvey 2020** | Low | Low | Low | Low | Low | Low | Low | Low |
| **Wilding 2021** | Low | Low | Low | Low | Low | Low | Unclear | Low |
| **Davies 2021** | Low | Low | Low | Low | Low | Low | Low | Low |
| **Wadden 2021** | Low | Low | Low | Low | Low | Low | Low | Low |
| **Garvey 2022** | Low | Low | Low | Low | Low | Low | Low | Low |
| **Kadowaki 2022** | Low | Low | Low | Low | Low | Low | Unclear | Low |
| **Mu 2024** | Low | Low | Low | Low | Low | Low | Unclear | Low |
| **Rubino 2022** | Low | Low | Low | Low | Low | Low | Unclear | Low |
| **Bliddal 2024** | Low | Low | Low | Low | Low | Low | Unclear | Low |
| **McGowan 2024** | Low | Low | Low | Low | Low | Low | Unclear | Low |
| **Wharton 2025^b^** | Low | Low | Low | Low | Low | Low | Low | Low |
| **Lingvay 2025** | Low | Low | Low | Low | Low | Low | Low | Low |
| **Kosiborod 2023** | Low | Low | Low | Low | Low | Low | Unclear | Low |
| **Kosiborod 2024** | Low | Low | Low | Low | Low | Low | Unclear | Low |
| **Wharton 2025** | Low | Low | Low | Low | Low | Low | Unclear | Low |

**Table S6.** P value from Egger’s test for seven cardiometabolic risk factors in the network meta‑analysis (NMA)

# Adults with obesity or overweight

| **Outcomes** | **Included studies** | **P value for Egger test** |
| --- | --- | --- |
| TBWL% | 18 | 0.812 |
| WC | 11 | 0.506 |
| HbA1c | 11 | 0.388 |
| SBP | 11 | 0.692 |
| TG | 11 | 0.120 |
| HDL-C | 11 | 0.389 |
| LDL-C | 11 | 0.069 |

1. Adults with obesity or overweight without T2D

| **Outcomes** | **Included studies** | **P value for Egger test** |
| --- | --- | --- |
| TBWL% | 11 | 0.960 |
| WC | 8 | 0.611 |
| HbA1c | 7 | 0.799 |
| SBP | 7 | 0.527 |
| TG | 7 | 0.559 |
| HDL-C | 7 | 0.322 |
| LDL-C | 7 | 0.178 |

1. Adults with obesity or overweight with T2D

| **Outcomes** | **Included studies** | **P value for Egger test** |
| --- | --- | --- |
| TBWL% | 6 | 0.453 |
| WC | 4 | NA† |
| HbA1c | 4 | NA† |
| SBP | 3 | NA† |
| TG | 3 | NA† |
| HDL-C | 3 | NA† |
| LDL-C | 3 | NA† |

**Abbreviation:** TBWL%, total body weight loss percentage; WC, waist circumference; HbA1c, haemoglobin A1c; SBP, systolic blood pressure; TG, triglyceride; HDL-C, high-density lipoprotein cholesterol; LDL-C, low-density lipoprotein cholesterol; T2D, type 2 diabetes.

† Egger's test was not performed when fewer than five studies were available because of insufficient power for reliable detection

**Table S7.** League table of direct and indirect comparisons among placebo and active treatments for adults with overweight or obesity in the network meta-analysis (NMA)

1. TBWL%

|  | **Liraglutide 1.8 mg** | **Liraglutide 3.0 mg** | **Orforglipron 12 mg** | **Orforglipron 36 mg** | **Orforglipron 6 mg** | **Placebo** | **Semaglutide 1.7 mg** | **Semaglutide 2.4 mg** | **Semaglutide 7.2 mg** |
| --- | --- | --- | --- | --- | --- | --- | --- | --- | --- |
| **Liraglutide 1.8 mg** | **Liraglutide 1.8 mg** | 1.35 (-3.42, 6.12) | . | . | . | -2.62 (-7.42, 2.18) | . | . | . |
| **Liraglutide 3.0 mg** | 1.38 (-2.17, 4.93) | **Liraglutide 3.0 mg** | . | . | . | -4.60 (-7.10, -2.10) | . | 10.50 (5.25, 15.75) | . |
| **Orforglipron 12 mg** | 4.35 (-0.55, 9.25) | 2.96 (-1.04, 6.97) | **Orforglipron 12 mg** | . | . | -7.00 (-10.38, -3.63) | . | . | . |
| **Orforglipron 36 mg** | 7.25 (2.35, 12.16) | 5.87 (1.86, 9.88) | 2.91 (-1.87, 7.69) | **Orforglipron 36 mg** | . | -9.91 (-13.29, -6.53) | . | . | . |
| **Orforglipron 6 mg** | 2.45 (-2.44, 7.34) | 1.06 (-2.93, 5.06) | -1.90 (-6.66, 2.87) | -4.81 (-9.58, -0.04) | **Orforglipron 6 mg** | -5.10 (-8.47, -1.74) | . | . | . |
| **Placebo** | -2.65 (-6.21, 0.90) | -4.04 (-6.19, -1.89) | -7.00 (-10.38, -3.63) | -9.91 (-13.29, -6.53) | -5.10 (-8.47, -1.74) | **Placebo** | 7.89 (2.73, 13.05) | 11.98 (10.48, 13.48) | 14.42 (10.87, 17.97) |
| **Semaglutide 1.7 mg** | 5.24 (-1.03, 11.50) | 3.85 (-1.74, 9.44) | 0.89 (-5.28, 7.05) | -2.02 (-8.19, 4.15) | 2.79 (-3.37, 8.94) | 7.89 (2.73, 13.05) | **Semaglutide 1.7 mg** | . | . |
| **Semaglutide 2.4 mg** | 9.44 (5.67, 13.22) | 8.06 (5.62, 10.49) | 5.09 (1.44, 8.75) | 2.19 (-1.47, 5.84) | 6.99 (3.35, 10.63) | 12.10 (10.70, 13.49) | 4.21 (-1.14, 9.55) | **Semaglutide 2.4 mg** | 3.30 (-0.24, 6.84) |
| **Semaglutide 7.2 mg** | 12.25 (7.87, 16.64) | 10.87 (7.54, 14.20) | 7.90 (3.64, 12.17) | 5.00 (0.73, 9.26) | 9.80 (5.55, 14.06) | 14.91 (12.30, 17.51) | 7.02 (1.24, 12.79) | 2.81 (0.21, 5.41) | **Semaglutide 7.2 mg** |

| 1. WC | |  |  |  |  |  |  |  |
| --- | --- | --- | --- | --- | --- | --- | --- | --- |
|  | **Liraglutide 3.0 mg** | **Orforglipron 12 mg** | **Orforglipron 36 mg** | **Orforglipron 6 mg** | **Placebo** | **Semaglutide 1.7 mg** | **Semaglutide 2.4 mg** | **Semaglutide 7.2 mg** |
| **Liraglutide 3.0 mg** | **Liraglutide 3.0 mg** | . | . | . | -3.40 (-6.19, -0.61) | . | 7.90 (3.66, 12.14) | . |
| **Orforglipron 12 mg** | 2.89 (-0.62, 6.41) | **Orforglipron 12 mg** | . | . | -5.71 (-8.30, -3.12) | . | . | . |
| **Orforglipron 36 mg** | 4.95 (1.43, 8.47) | 2.05 (-1.62, 5.73) | **Orforglipron 36 mg** | . | -7.77 (-10.36, -5.17) | . | . | . |
| **Orforglipron 6 mg** | 1.39 (-2.12, 4.90) | -1.50 (-5.16, 2.16) | -3.56 (-7.22, 0.11) | **Orforglipron 6 mg** | -4.21 (-6.80, -1.63) | . | . | . |
| **Placebo** | -2.82 (-5.19, -0.45) | -5.71 (-8.30, -3.12) | -7.77 (-10.36, -5.17) | -4.21 (-6.80, -1.63) | **Placebo** | 6.30 (2.25, 10.35) | 9.36 (7.77, 10.96) | 11.01 (8.15, 13.88) |
| **Semaglutide 1.7 mg** | 3.48 (-1.21, 8.17) | 0.59 (-4.22, 5.40) | -1.47 (-6.28, 3.34) | 2.09 (-2.72, 6.89) | 6.30 (2.25, 10.35) | **Semaglutide 1.7 mg** | . | . |
| **Semaglutide 2.4 mg** | 6.56 (4.03, 9.10) | 3.67 (0.71, 6.63) | 1.62 (-1.34, 4.58) | 5.17 (2.22, 8.12) | 9.38 (7.96, 10.81) | 3.08 (-1.21, 7.38) | **Semaglutide 2.4 mg** | 2.72 (-0.13, 5.58) |
| **Semaglutide 7.2 mg** | 8.74 (5.64, 11.84) | 5.85 (2.48, 9.21) | 3.79 (0.43, 7.16) | 7.35 (3.99, 10.71) | 11.56 (9.42, 13.70) | 5.26 (0.68, 9.84) | 2.18 (0.03, 4.32) | **Semaglutide 7.2 mg** |
| 1. HbA1c |  |  |  |  |  |  |  |  |
|  | **Liraglutide 3.0 mg** | **Orforglipron 12 mg** | **Orforglipron 36 mg** | **Orforglipron 6 mg** | **Placebo** | **Semaglutide 1.7 mg** | **Semaglutide 2.4 mg** | **Semaglutide 7.2 mg** |
| **Liraglutide 3.0 mg** | **Liraglutide 3.0 mg** | . | . | . | -0.60 (-1.34, 0.14) | . | 0.20 (-0.51, 0.91) | . |
| **Orforglipron 12 mg** | 0.29 (-0.45, 1.03) | **Orforglipron 12 mg** | . | . | -0.86 (-1.37, -0.35) | . | . | . |
| **Orforglipron 36 mg** | 0.42 (-0.32, 1.16) | 0.13 (-0.59, 0.85) | **Orforglipron 36 mg** | . | -0.98 (-1.49, -0.47) | . | . | . |
| **Orforglipron 6 mg** | 0.14 (-0.60, 0.88) | -0.15 (-0.87, 0.57) | -0.28 (-1.00, 0.45) | **Orforglipron 6 mg** | -0.71 (-1.22, -0.20) | . | . | . |
| **Placebo** | -0.57 (-1.10, -0.03) | -0.86 (-1.37, -0.35) | -0.98 (-1.49, -0.47) | -0.71 (-1.22, -0.20) | **Placebo** | 0.89 (0.16, 1.62) | 0.68 (0.36, 1.00) | 1.16 (0.64, 1.68) |
| **Semaglutide 1.7 mg** | 0.32 (-0.58, 1.23) | 0.03 (-0.86, 0.93) | -0.09 (-0.99, 0.80) | 0.18 (-0.71, 1.08) | 0.89 (0.16, 1.62) | **Semaglutide 1.7 mg** | . | . |
| **Semaglutide 2.4 mg** | 0.17 (-0.36, 0.70) | -0.12 (-0.70, 0.46) | -0.25 (-0.83, 0.34) | 0.03 (-0.56, 0.61) | 0.74 (0.45, 1.02) | -0.15 (-0.94, 0.63) | **Semaglutide 2.4 mg** | 0.16 (-0.35, 0.68) |
| **Semaglutide 7.2 mg** | 0.46 (-0.17, 1.09) | 0.17 (-0.47, 0.82) | 0.05 (-0.60, 0.69) | 0.32 (-0.32, 0.97) | 1.03 (0.64, 1.42) | 0.14 (-0.69, 0.97) | 0.29 (-0.10, 0.69) | **Semaglutide 7.2 mg** |
| 1. SBP |  |  |  |  |  |  |  |  |
|  | **Liraglutide 3.0 mg** | **Orforglipron 12 mg** | **Orforglipron 36 mg** | **Orforglipron 6 mg** | **Placebo** | **Semaglutide 1.7 mg** | **Semaglutide 2.4 mg** | **Semaglutide 7.2 mg** |
| **Liraglutide 3.0 mg** | **Liraglutide 3.0 mg** | . | . | . | . | . | 1.20 (-2.46, 4.86) | . |
| **Orforglipron 12 mg** | 0.57 (-3.47, 4.61) | **Orforglipron 12 mg** | . | . | -4.30 (-5.70, -2.90) | . | . | . |
| **Orforglipron 36 mg** | 1.57 (-2.48, 5.62) | 1.00 (-1.00, 3.00) | **Orforglipron 36 mg** | . | -5.30 (-6.73, -3.87) | . | . | . |
| **Orforglipron 6 mg** | 0.39 (-3.65, 4.44) | -0.18 (-2.16, 1.81) | -1.18 (-3.18, 0.83) | **Orforglipron 6 mg** | -4.12 (-5.53, -2.71) | . | . | . |
| **Placebo** | -3.73 (-7.52, 0.06) | -4.30 (-5.70, -2.90) | -5.30 (-6.73, -3.87) | -4.12 (-5.53, -2.71) | **Placebo** | 6.02 (2.25, 9.79) | 4.98 (3.92, 6.03) | 6.13 (4.16, 8.10) |
| **Semaglutide 1.7 mg** | 2.29 (-3.06, 7.64) | 1.72 (-2.30, 5.75) | 0.72 (-3.31, 4.76) | 1.90 (-2.13, 5.92) | 6.02 (2.25, 9.79) | **Semaglutide 1.7 mg** | . | . |
| **Semaglutide 2.4 mg** | 1.20 (-2.46, 4.86) | 0.63 (-1.08, 2.34) | -0.37 (-2.10, 1.36) | 0.81 (-0.91, 2.52) | 4.93 (3.95, 5.91) | -1.09 (-4.99, 2.81) | **Semaglutide 2.4 mg** | 1.54 (-0.38, 3.46) |
| **Semaglutide 7.2 mg** | 2.57 (-1.37, 6.51) | 2.01 (-0.02, 4.03) | 1.01 (-1.04, 3.05) | 2.18 (0.15, 4.21) | 6.30 (4.84, 7.77) | 0.28 (-3.76, 4.33) | 1.37 (-0.08, 2.83) | **Semaglutide 7.2 mg** |
| 1. TG |  |  |  |  |  |  |  |  |
|  | **Liraglutide 3.0 mg** | **Orforglipron 12 mg** | **Orforglipron 36 mg** | **Orforglipron 6 mg** | **Placebo** | **Semaglutide 1.7 mg** | **Semaglutide 2.4 mg** | **Semaglutide 7.2 mg** |
| **Liraglutide 3.0 mg** | **Liraglutide 3.0 mg** | -0.02 (-11.24, 12.61) | 7.54 (-4.49, 21.08) | -2.44 (-13.37, 9.87) | -11.34 (-20.37, -1.28) | 18.21 (0.23, 39.42) | 10.50 (-0.21, 22.35) | 20.13 (7.28, 34.51) |
| **Orforglipron 12 mg** | 0.02 (-11.20, 12.66) | **Orforglipron 12 mg** | 7.56 (0.13, 15.54) | -2.42 (-9.19, 4.86) | -11.32 (-15.73, -6.68) | 18.24 (3.29, 35.35) | 10.52 (3.95, 17.52) | 20.15 (11.87, 29.06) |
| **Orforglipron 36 mg** | -7.01 (-17.41, 4.70) | -7.03 (-13.45, -0.13) | **Orforglipron 36 mg** | -9.28 (-15.52, -2.57) | -17.55 (-21.59, -13.31) | 9.93 (-3.94, 25.80) | 2.75 (-3.29, 9.18) | 11.71 (4.07, 19.91) |
| **Orforglipron 6 mg** | 2.50 (-8.98, 15.44) | 2.48 (-4.63, 10.12) | 10.23 (2.64, 18.38) | **Orforglipron 6 mg** | -9.12 (-13.61, -4.39) | 21.17 (5.86, 38.69) | 13.26 (6.55, 20.40) | 23.13 (14.67, 32.23) |
| **Placebo** | 12.79 (1.30, 25.59) | 12.76 (7.15, 18.67) | 21.29 (15.36, 27.53) | 10.04 (4.59, 15.76) | **Placebo** | 33.33 (17.65, 51.11) | 24.63 (20.46, 28.95) | 35.49 (28.88, 42.44) |
| **Semaglutide 1.7 mg** | -15.41 (-28.27, -0.23) | -15.43 (-26.12, -3.19) | -9.03 (-20.51, 4.10) | -17.47 (-27.90, -5.54) | -25.00 (-33.82, -15.00) | **Semaglutide 1.7 mg** | -6.53 (-17.90, 6.42) | 1.62 (-11.20, 16.28) |
| **Semaglutide 2.4 mg** | -9.50 (-18.27, 0.21) | -9.52 (-14.91, -3.80) | -2.68 (-8.41, 3.40) | -11.71 (-16.94, -6.15) | -19.76 (-22.45, -16.98) | 6.98 (-6.03, 21.80) | **Semaglutide 2.4 mg** | 8.71 (3.51, 14.18) |
| **Semaglutide 7.2 mg** | -16.75 (-25.66, -6.78) | -16.77 (-22.51, -10.61) | -10.48 (-16.60, -3.91) | -18.79 (-24.37, -12.79) | -26.19 (-29.80, -22.41) | -1.59 (-14.00, 12.61) | -8.02 (-12.42, -3.39) | **Semaglutide 7.2 mg** |
| 1. HDL-C |  |  |  |  |  |  |  |  |
|  | **Liraglutide 3.0 mg** | **Orforglipron 12 mg** | **Orforglipron 36 mg** | **Orforglipron 6 mg** | **Placebo** | **Semaglutide 1.7 mg** | **Semaglutide 2.4 mg** | **Semaglutide 7.2 mg** |
| **Liraglutide 3.0 mg** | **Liraglutide 3.0 mg** | 2.09 (-2.85, 7.28) | -0.35 (-5.17, 4.71) | 2.87 (-2.10, 8.09) | 5.43 (0.75, 10.33) | 4.38 (-2.02, 11.21) | 2.88 (-1.51, 7.47) | -0.22 (-4.87, 4.67) |
| **Orforglipron 12 mg** | -2.05 (-6.78, 2.93) | **Orforglipron 12 mg** | -2.39 (-5.10, 0.39) | 0.77 (-2.01, 3.62) | 3.27 (1.24, 5.34) | 2.25 (-2.58, 7.32) | 0.78 (-1.57, 3.17) | -2.26 (-4.97, 0.53) |
| **Orforglipron 36 mg** | 0.35 (-4.50, 5.46) | 2.45 (-0.39, 5.37) | **Orforglipron 36 mg** | 3.24 (0.39, 6.16) | 5.80 (3.72, 7.92) | 4.76 (-0.19, 9.95) | 3.25 (0.85, 5.70) | 0.14 (-2.64, 2.99) |
| **Orforglipron 6 mg** | -2.79 (-7.49, 2.14) | -0.76 (-3.50, 2.05) | -3.14 (-5.80, -0.39) | **Orforglipron 6 mg** | 2.49 (0.49, 4.52) | 1.47 (-3.32, 6.49) | 0.01 (-2.30, 2.37) | -3.00 (-5.68, -0.25) |
| **Placebo** | -5.15 (-9.36, -0.74) | -3.17 (-5.07, -1.23) | -5.48 (-7.34, -3.59) | -2.42 (-4.32, -0.49) | **Placebo** | -0.99 (-5.27, 3.48) | -2.42 (-3.64, -1.18) | -5.35 (-7.22, -3.45) |
| **Semaglutide 1.7 mg** | -4.20 (-10.08, 2.06) | -2.20 (-6.82, 2.65) | -4.54 (-9.05, 0.19) | -1.45 (-6.10, 3.43) | 1.00 (-3.36, 5.56) | **Semaglutide 1.7 mg** | -1.44 (-5.86, 3.19) | -4.41 (-8.93, 0.34) |
| **Semaglutide 2.4 mg** | -2.80 (-6.95, 1.54) | -0.77 (-3.08, 1.59) | -3.14 (-5.40, -0.84) | -0.01 (-2.31, 2.35) | 2.48 (1.19, 3.78) | 1.46 (-3.09, 6.23) | **Semaglutide 2.4 mg** | -3.01 (-4.88, -1.10) |
| **Semaglutide 7.2 mg** | 0.22 (-4.46, 5.12) | 2.31 (-0.53, 5.23) | -0.14 (-2.91, 2.71) | 3.09 (0.25, 6.02) | 5.66 (3.58, 7.78) | 4.61 (-0.33, 9.80) | 3.10 (1.11, 5.13) | **Semaglutide 7.2 mg** |
| 1. LDL-C |  |  |  |  |  |  |  |  |
|  | **Liraglutide 3.0 mg** | **Orforglipron 12 mg** | **Orforglipron 36 mg** | **Orforglipron 6 mg** | **Placebo** | **Semaglutide 1.7 mg** | **Semaglutide 2.4 mg** | **Semaglutide 7.2 mg** |
| **Liraglutide 3.0 mg** | **Liraglutide 3.0 mg** | 6.53 (-2.93, 16.90) | 5.92 (-3.49, 16.25) | 3.96 (-5.31, 14.13) | 2.65 (-5.35, 11.33) | 10.38 (-1.78, 24.04) | 9.17 (1.10, 17.88) | 6.39 (-2.53, 16.13) |
| **Orforglipron 12 mg** | -6.13 (-14.46, 3.02) | **Orforglipron 12 mg** | -0.57 (-6.75, 6.03) | -2.41 (-8.53, 4.11) | -3.64 (-7.90, 0.83) | 3.62 (-5.80, 13.98) | 2.48 (-2.75, 7.99) | -0.13 (-6.13, 6.26) |
| **Orforglipron 36 mg** | -5.59 (-13.98, 3.62) | 0.57 (-5.68, 7.24) | **Orforglipron 36 mg** | -1.85 (-8.02, 4.73) | -3.08 (-7.40, 1.43) | 4.21 (-5.27, 14.64) | 3.07 (-2.21, 8.64) | 0.45 (-5.61, 6.89) |
| **Orforglipron 6 mg** | -3.81 (-12.38, 5.61) | 2.47 (-3.95, 9.32) | 1.89 (-4.51, 8.72) | **Orforglipron 6 mg** | -1.25 (-5.72, 3.42) | 6.18 (-3.52, 16.85) | 5.01 (-0.43, 10.75) | 2.34 (-3.88, 8.97) |
| **Placebo** | -2.58 (-10.18, 5.65) | 3.77 (-0.82, 8.58) | 3.18 (-1.41, 7.99) | 1.27 (-3.30, 6.06) | **Placebo** | 7.53 (-1.12, 16.93) | 6.35 (3.58, 9.19) | 3.64 (-0.66, 8.13) |
| **Semaglutide 1.7 mg** | -9.40 (-19.38, 1.81) | -3.49 (-12.26, 6.16) | -4.04 (-12.77, 5.57) | -5.82 (-14.42, 3.65) | -7.00 (-14.48, 1.13) | **Semaglutide 1.7 mg** | -1.10 (-9.42, 7.99) | -3.61 (-12.25, 5.88) |
| **Semaglutide 2.4 mg** | -8.40 (-15.17, -1.09) | -2.42 (-7.40, 2.83) | -2.98 (-7.95, 2.26) | -4.78 (-9.71, 0.43) | -5.97 (-8.41, -3.46) | 1.11 (-7.40, 10.39) | **Semaglutide 2.4 mg** | -2.54 (-6.57, 1.65) |
| **Semaglutide 7.2 mg** | -6.01 (-13.89, 2.60) | 0.13 (-5.89, 6.53) | -0.44 (-6.45, 5.94) | -2.29 (-8.23, 4.03) | -3.51 (-7.52, 0.66) | 3.75 (-5.55, 13.97) | 2.61 (-1.63, 7.03) | **Semaglutide 7.2 mg** |

**Abbreviation:** TBWL%, total body weight loss percentage; WC, waist circumference; HbA1c, haemoglobin A1c; SBP, systolic blood pressure; TG, triglyceride; HDL-C, high-density lipoprotein cholesterol; LDL-C, low-density lipoprotein cholesterol.

**Note:** In the league table, the upper triangular cells show the effect estimates (mean difference, with 95% confidence intervals) for the column treatment versus the row treatment, while the lower triangular cells show the corresponding estimates for the row treatment versus the column treatment. Positive values indicate a more favourable effect for the column treatment relative to the row treatment, and negative values indicate a more favourable effect for the row treatment relative to the column treatment.

# **Table S8.** GRADE checklist

| **Compound** | **Certainty assessment** | | | | | **Effect, MD (95% CI)** | **Overall certainty of evidence** |
| --- | --- | --- | --- | --- | --- | --- | --- |
|  | **Risk of bias** | **Inconsistency** | **Indirectness** | **Imprecision** | **Publication bias** |  |  |
| **Endpoint TBWL%** | | | | | | | |
| Semaglutide 7.2 mg | not serious | serious^a^ | not serious | not serious | not serious | **-14.91 (-17.51 to -12.30)** | ⨁⨁⨁◯ Moderate |
| Semaglutide 2.4 mg | not serious | serious | not serious | not serious | not serious | **-12.1 (-13.49 to -10.70)** | ⨁⨁⨁◯ Moderate |
| Semaglutide 1.7 mg | not serious | serious | not serious | serious^b^ | not serious | **-7.89 (-13.05 to -2.73)** | ⨁⨁◯◯ Low |
| Orforglipron 36 mg | not serious | serious | not serious | not serious | not serious | **-9.91(-13.29, -6.53)** | ⨁⨁⨁◯ Moderate |
| Orforglipron 12 mg | not serious | serious | not serious | not serious | not serious | **-7.00 (-10.38, -3.63)** | ⨁⨁⨁◯ Moderate |
| Orforglipron 6 mg | not serious | serious | not serious | not serious | not serious | **-5.10 (-8.47, -1.74)** | ⨁⨁⨁◯ Moderate |
| Liraglutide 3.0 mg | not serious | serious | not serious | not serious | not serious | **-4.04 (-6.19, -1.89)** | ⨁⨁⨁◯ Moderate |
| Liraglutide 1.8 mg | not serious | serious | not serious | serious^b^ | not serious | **-2.65 (-6.21, 0.90)** | ⨁⨁◯◯ Low |
| **Endpoint WC** | | | | | | | |
| Semaglutide 7.2 mg | not serious | serious | not serious | not serious | not serious | **-11.56 (-13.70, -9.42)** | ⨁⨁⨁◯ Moderate |
| Semaglutide 2.4 mg | not serious | serious | not serious | not serious | not serious | **-9.38 (-10.81, -7.96)** | ⨁⨁⨁◯ Moderate |
| Semaglutide 1.7 mg | not serious | serious | not serious | serious^b^ | not serious | **-6.30 (-10.35, -2.25)** | ⨁⨁◯◯ Low |
| Orforglipron 36 mg | not serious | serious | not serious | serious^c^ | not serious | **-7.77 (-10.36, -5.17)** | ⨁⨁⨁◯ Moderate |
| Orforglipron 12 mg | not serious | serious | not serious | not serious | not serious | **-5.71 (-8.30, -3.12)** | ⨁⨁⨁◯ Moderate |
| Orforglipron 6 mg | not serious | serious | not serious | not serious | not serious | **-4.21 (-6.80, -1.63)** | ⨁⨁⨁◯ Moderate |
| Liraglutide 3.0 mg | not serious | serious | not serious | not serious | not serious | **-2.82 (-5.19, -0.45)** | ⨁⨁⨁◯ Moderate |
| **Endpoint HbA1c** | | | | | | | |
| Semaglutide 7.2 mg | not serious | serious | not serious | not serious | not serious | **-1.03 (-1.42, -0.64)** | ⨁⨁⨁◯ Moderate |
| Semaglutide 2.4 mg | not serious | serious | not serious | not serious | not serious | **-0.74 (-1.02, -0.45)** | ⨁⨁⨁◯ Moderate |
| Semaglutide 1.7 mg | not serious | serious | not serious | serious^b^ | not serious | **-0.89 (-1.62, -0.16)** | ⨁⨁◯◯ Low |
| Orforglipron 36 mg | not serious | serious | not serious | not serious | not serious | **-0.98 (-1.49, -0.47)** | ⨁⨁⨁◯ Moderate |
| Orforglipron 12 mg | not serious | serious | not serious | not serious | not serious | **-0.86 (-1.37, -0.35)** | ⨁⨁⨁◯ Moderate |
| Orforglipron 6 mg | not serious | serious | not serious | not serious | not serious | **-0.71 (-1.22, -0.20)** | ⨁⨁⨁◯ Moderate |
| Liraglutide 3.0 mg | not serious | serious | not serious | not serious | not serious | **-0.57 (-1.10, -0.03)** | ⨁⨁⨁◯ Moderate |
| **Endpoint SBP** | | | | | | | |
| Semaglutide 7.2 mg | not serious | not serious | not serious | not serious | not serious | **-6.30 (-7.77, -4.84)** | ⨁⨁⨁◯ Moderate |
| Semaglutide 2.4 mg | not serious | not serious | not serious | not serious | not serious | **-4.93 (-5.91, -3.95)** | ⨁⨁⨁◯ Moderate |
| Semaglutide 1.7 mg | not serious | not serious | not serious | serious^b^ | not serious | **-6.02 (-9.79, -2.25)** | ⨁⨁◯◯ Low |
| Orforglipron 36 mg | not serious | not serious | not serious | not serious | not serious | **-5.30 (-6.73, -3.87)** | ⨁⨁⨁◯ Moderate |
| Orforglipron 12 mg | not serious | not serious | not serious | not serious | not serious | **-4.30 (-5.70, -2.90)** | ⨁⨁⨁◯ Moderate |
| Orforglipron 6 mg | not serious | not serious | not serious | not serious | not serious | **-4.12 (-5.53, -2.71)** | ⨁⨁⨁◯ Moderate |
| Liraglutide 3.0 mg | not serious | not serious | not serious | serious^b^ | not serious | **-3.73 (-7.52, 0.06)** | ⨁⨁◯◯ Low |
| **Endpoint TG** | | | | | | | |
| Semaglutide 7.2 mg | not serious | not serious | not serious | not serious | not serious | **-26.19 (-29.80, -22.41)** | ⨁⨁⨁◯ Moderate |
| Semaglutide 2.4 mg | not serious | not serious | not serious | not serious | not serious | **-19.76 (-22.45, -16.98)** | ⨁⨁⨁◯ Moderate |
| Semaglutide 1.7 mg | not serious | not serious | not serious | serious^b^ | not serious | **-25.00 (-33.82, -15.00)** | ⨁⨁◯◯ Low |
| Orforglipron 36 mg | not serious | not serious | not serious | not serious | not serious | **-17.55 (-21.59, -13.31)** | ⨁⨁⨁◯ Moderate |
| Orforglipron 12 mg | not serious | not serious | not serious | not serious | not serious | **-11.32 (-15.73, -6.68)** | ⨁⨁⨁◯ Moderate |
| Orforglipron 6 mg | not serious | not serious | not serious | not serious | not serious | **-9.12 (-13.61, -4.39)** | ⨁⨁⨁◯ Moderate |
| Liraglutide 3.0 mg | not serious | not serious | not serious | serious^b^ | not serious | **-11.34 (-20.37, -1.28)** | ⨁⨁◯◯ Low |
| **Endpoint HDL-C** | | | | | | | |
| Semaglutide 7.2 mg | not serious | not serious | not serious | not serious | not serious | **5.66 (3.58, 7.78)** | ⨁⨁⨁◯ Moderate |
| Semaglutide 2.4 mg | not serious | not serious | not serious | not serious | not serious | **2.48 (1.19, 3.78)** | ⨁⨁⨁◯ Moderate |
| Semaglutide 1.7 mg | not serious | not serious | not serious | serious^b^ | not serious | **1.00 (-3.36, 5.56)** | ⨁⨁◯◯ Low |
| Orforglipron 36 mg | not serious | not serious | not serious | not serious | not serious | **5.80 (3.72, 7.92)** | ⨁⨁⨁◯ Moderate |
| Orforglipron 12 mg | not serious | not serious | not serious | not serious | not serious | **3.27 (1.24, 5.34)** | ⨁⨁⨁◯ Moderate |
| Orforglipron 6 mg | not serious | not serious | not serious | not serious | not serious | **2.49 (0.49, 4.52)** | ⨁⨁⨁◯ Moderate |
| Liraglutide 3.0 mg | not serious | not serious | not serious | serious | not serious | **5.43 (0.75, 10.33)** | ⨁⨁⨁◯ Moderate |
| **Endpoint LDL-C** | | | | | | | |
| Semaglutide 7.2 mg | not serious | serious | not serious | serious | not serious | **-3.51 (-7.52, 0.66)** | ⨁⨁⨁◯ Moderate |
| Semaglutide 2.4 mg | not serious | serious | not serious | not serious | not serious | **-5.97 (-8.41, -3.46)** | ⨁⨁⨁◯ Moderate |
| Semaglutide 1.7 mg | not serious | serious | not serious | serious^b^ | not serious | **-7.00 (-14.48, 1.13)** | ⨁⨁⨁◯ Moderate |
| Orforglipron 36 mg | not serious | serious | not serious | not serious | not serious | **-3.08(-7.40, 1.43)** | ⨁⨁⨁◯ Moderate |
| Orforglipron 12 mg | not serious | serious | not serious | not serious | not serious | **-3.64 (-7.90, 0.83)** | ⨁⨁⨁◯ Moderate |
| Orforglipron 6 mg | not serious | serious | not serious | not serious | not serious | **-1.25 (-5.72, 3.42)** | ⨁⨁⨁◯ Moderate |
| Liraglutide 3.0 mg | not serious | serious | not serious | serious^b^ | not serious | **2.65 (-5.35, 11.33)** | ⨁⨁⨁◯ Moderate |

**Abbreviation:** MD, mean difference; CI, confidence interval; TBWL%, total body weight loss percentage; WC, waist circumference; HbA1c, haemoglobin A1c; SBP, systolic blood pressure; TG, triglyceride; HDL-C, high-density lipoprotein cholesterol; LDL-C, low-density lipoprotein cholesterol.

**Explanations:**

1. $I^{2}$> 50% (high heterogeneity)
2. Data coming from one single study
3. Wide variance of point estimates across studies

**Figure S1.** PRISMA flow diagram displaying results of the literature search

Total number of studies included (n=19)

Records excluded with reason (n=278)

Wrong population/indication (n = **170)**

Conference abstracts / non-full-text reports (n = **60)**

Ineligible study design (n = **19)**

Wrong intervention/comparator (n = **9)**

Insufficient sample size or duration (n = **16)**

Wrong or unreported outcomes (n = **4)**

Duplicated records removed (n=938)

Records excluded (n=3787)

Full text sought for eligibility (n=297)

Records screened by title/abstract (n=4084)

Studies from databases (n = 5022)

PubMed (n = 738)

Embase (n = 841)

CENTRAL (n = 3443)

**Figure S2.** Pairwise-origami plot summarizing multidimensional efficacy across seven cardiometabolic risk factors in adults with overweight or obesity

**
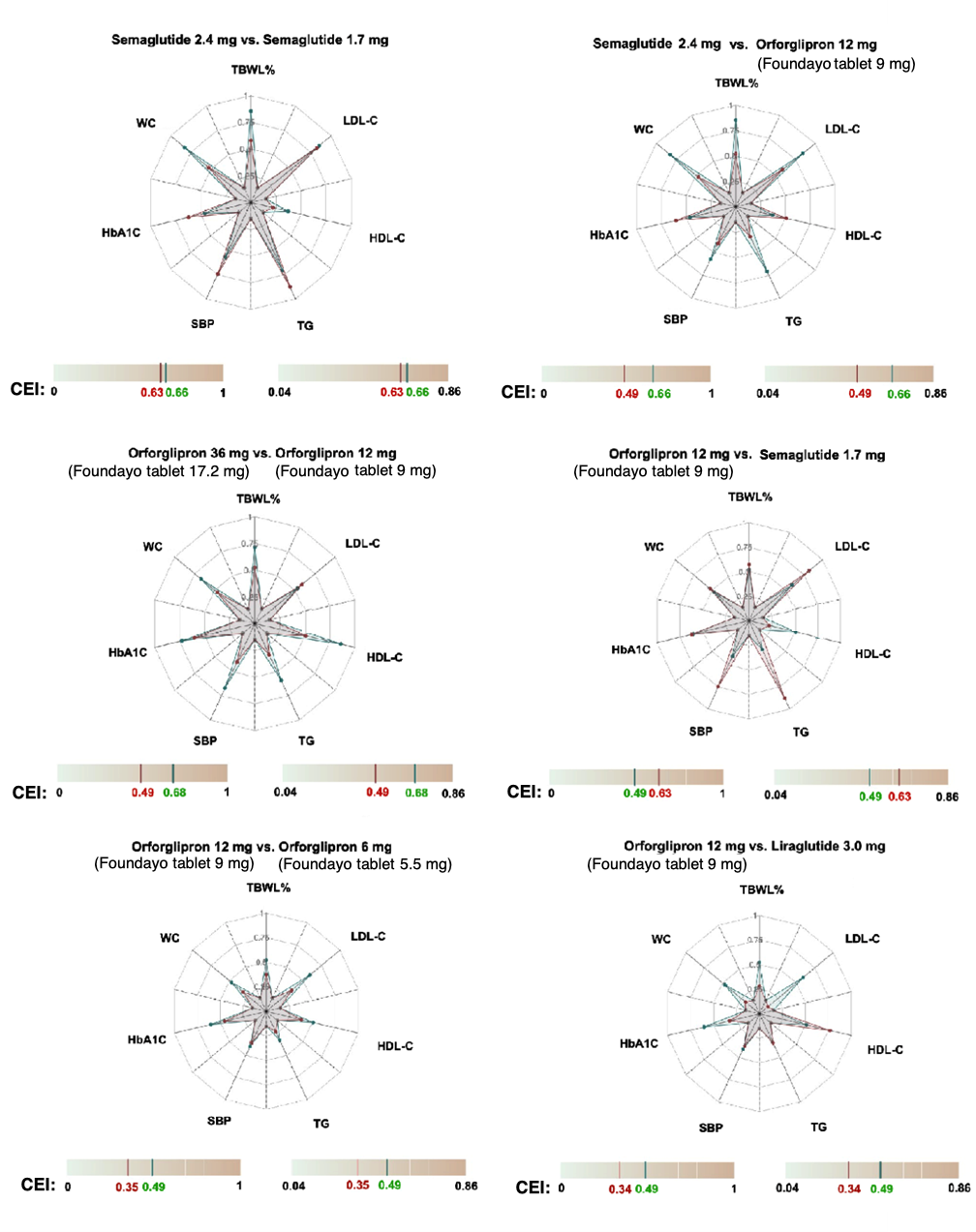
**

**Abbreviation:** HbA1c, haemoglobin A1c; HDL-C, high-density lipoprotein cholesterol; LDL-C, low-density lipoprotein cholesterol; SBP, systolic blood pressure; TBWL%, total body weight loss percentage; TG, triglyceride; WC, waist circumference.

**Note:** Origami plots visualize multidimensional cardiometabolic profiles using SUCRA rankings across seven outcomes (TBWL%, WC, HbA1c, SBP, TG, HDL-C, LDL-C). Each axis represents a SUCRA value, and the polygon area is normalized (0–1) to calculate the Cardiometabolic Efficacy Index (CEI), with larger areas indicating indicate more favourable overall profiles. In pairwise plots, the **green polygon** represents the primary treatment and the **red polygon** the comparator. The lower-left scale shows the normalized CEI (0–1), and the lower-right scale shows the absolute minimum and maximum polygon areas across all treatments in the network, with colour-coded values corresponding specifically to the two compared treatments.

**Figure S3.** Forest and network plots of cardiometabolic risk factors for glucagon-Like peptide-1 (GLP-1) receptor mono-agonist versus placebo in adults with overweight or obesity without T2D


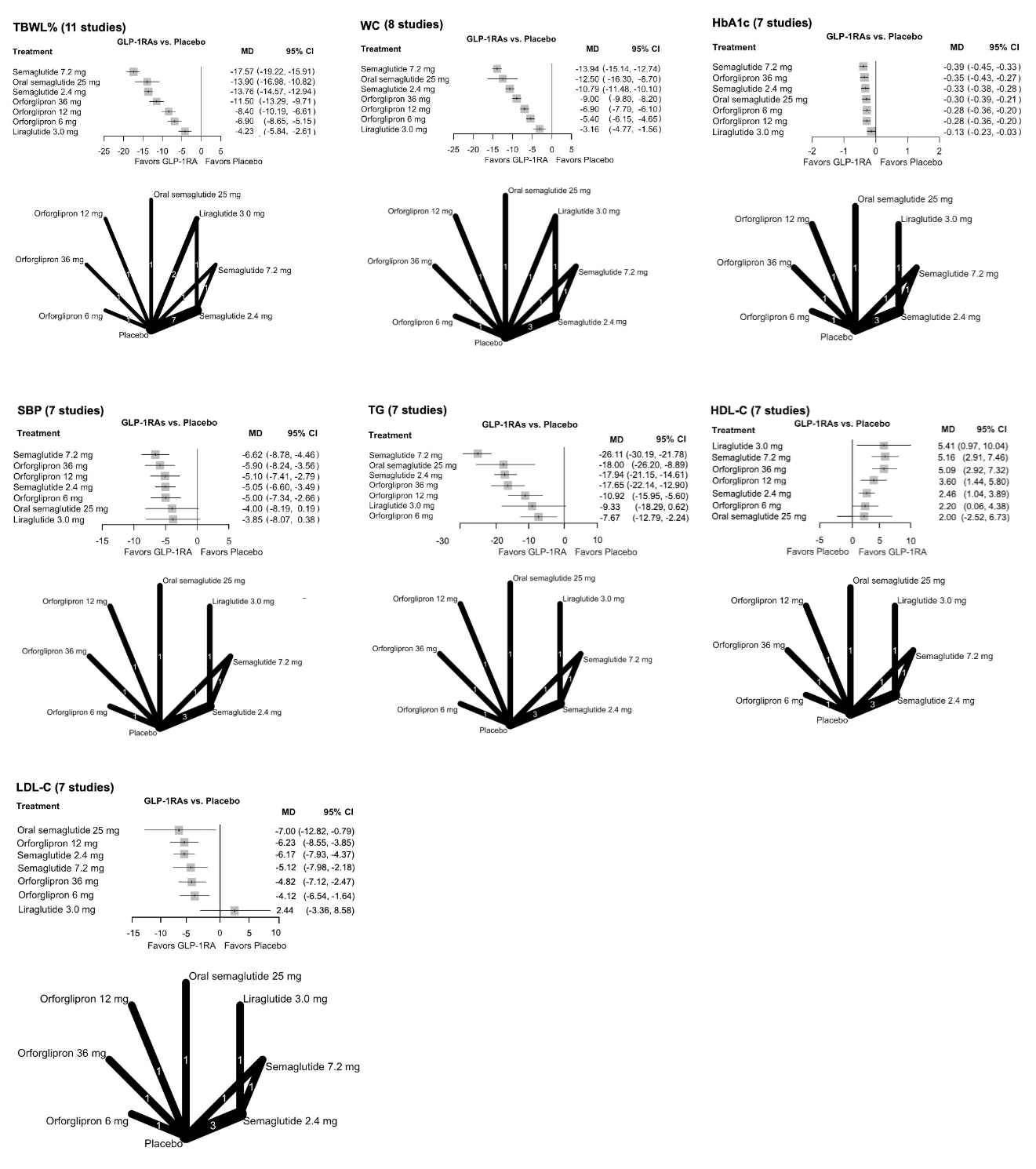


**Abbreviation:** HbA1c, haemoglobin A1c; HDL-C, high-density lipoprotein cholesterol; LDL-C, low-density lipoprotein cholesterol; SBP, systolic blood pressure; TBWL%, total body weight loss percentage; TG, triglyceride; WC, waist circumference.

**Note:** **Top panels:** Forest plots showing mean differences (MD) and 95% confidence intervals (CI) for each treatment versus placebo across seven cardiometabolic risk factors (TBWL%, WC, HbA1c, SBP, TG, HDL-C, and LDL-C).

**Bottom panels:** Network plots visualizing the evidence structure. Nodes represent specific treatment arms and lines (edges) connecting nodes indicate direct head-to-head clinical trials, with line thickness proportional to the number of studies informing each comparison (thicker lines indicate more direct evidence). Closed loops show comparisons supported by both direct and indirect evidence, enabling statistical assessment of network consistency.

**Figure S4.** Pairwise-origami plot summarizing multidimensional efficacy across seven cardiometabolic risk factors in adults with overweight or obesity without T2D

**
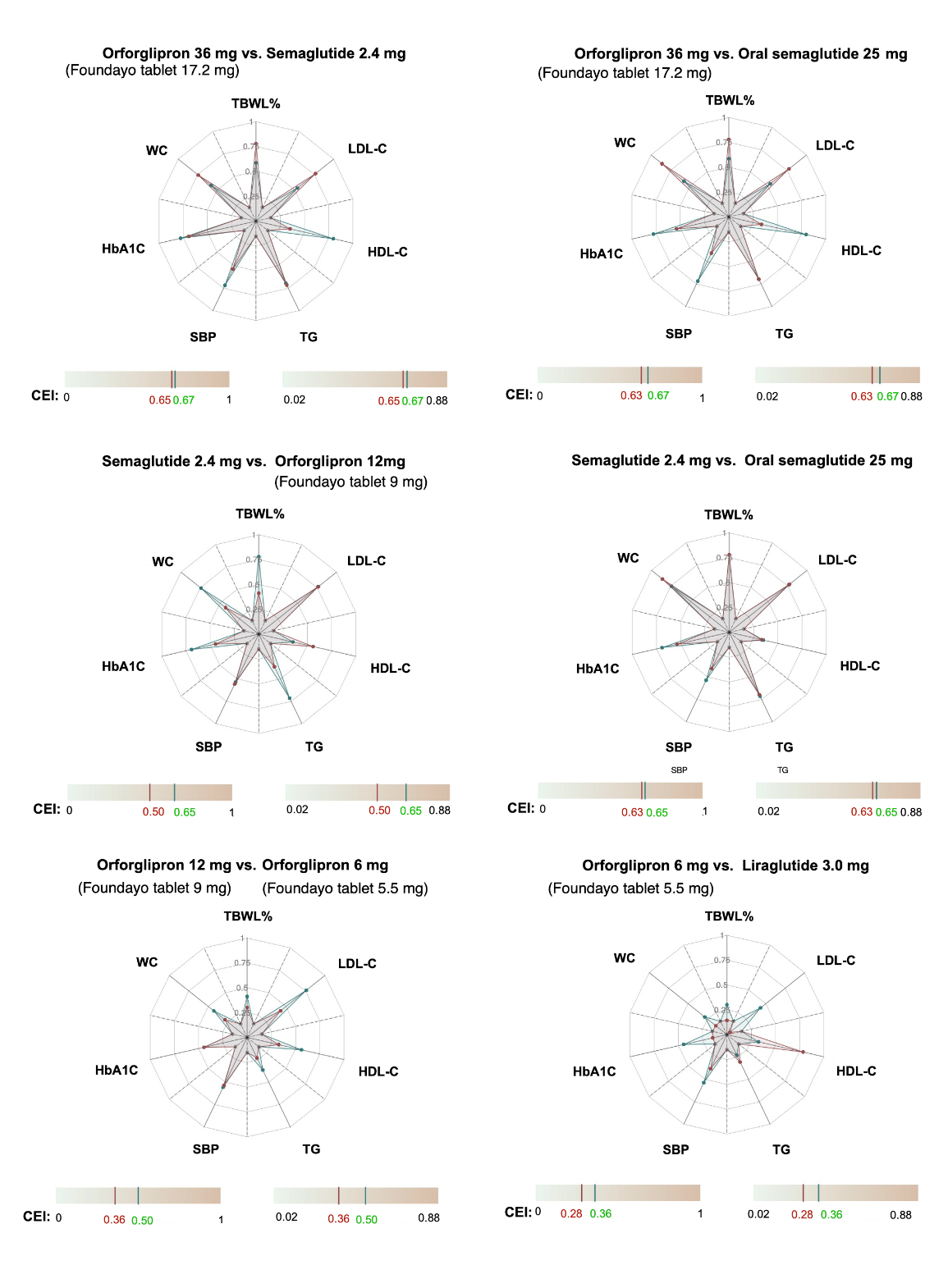
**

**Abbreviation:** HbA1c, haemoglobin A1c; HDL-C, high-density lipoprotein cholesterol; LDL-C, low-density lipoprotein cholesterol; SBP, systolic blood pressure; TBWL%, total body weight loss percentage; TG, triglyceride; WC, waist circumference.

**Note:** Origami plots visualize multidimensional cardiometabolic profiles using SUCRA rankings across seven outcomes (TBWL%, WC, HbA1c, SBP, TG, HDL-C, LDL-C). Each axis represents a SUCRA value, and the polygon area is normalized (0–1) to calculate the Cardiometabolic Efficacy Index (CEI), with larger areas indicating indicate more favourable overall profiles. In pairwise plots, the **green polygon** represents the primary treatment and the **red polygon** the comparator. The lower-left scale shows the normalized CEI (0–1), and the lower-right scale shows the absolute minimum and maximum polygon areas across all treatments in the network, with colour-coded values corresponding specifically to the two compared treatments.

**Figure S5.** Forest and network plots of cardiometabolic risk factors for glucagon-Like peptide-1 (GLP-1) receptor mono-agonist versus placebo in adults with overweight or obesity with T2D


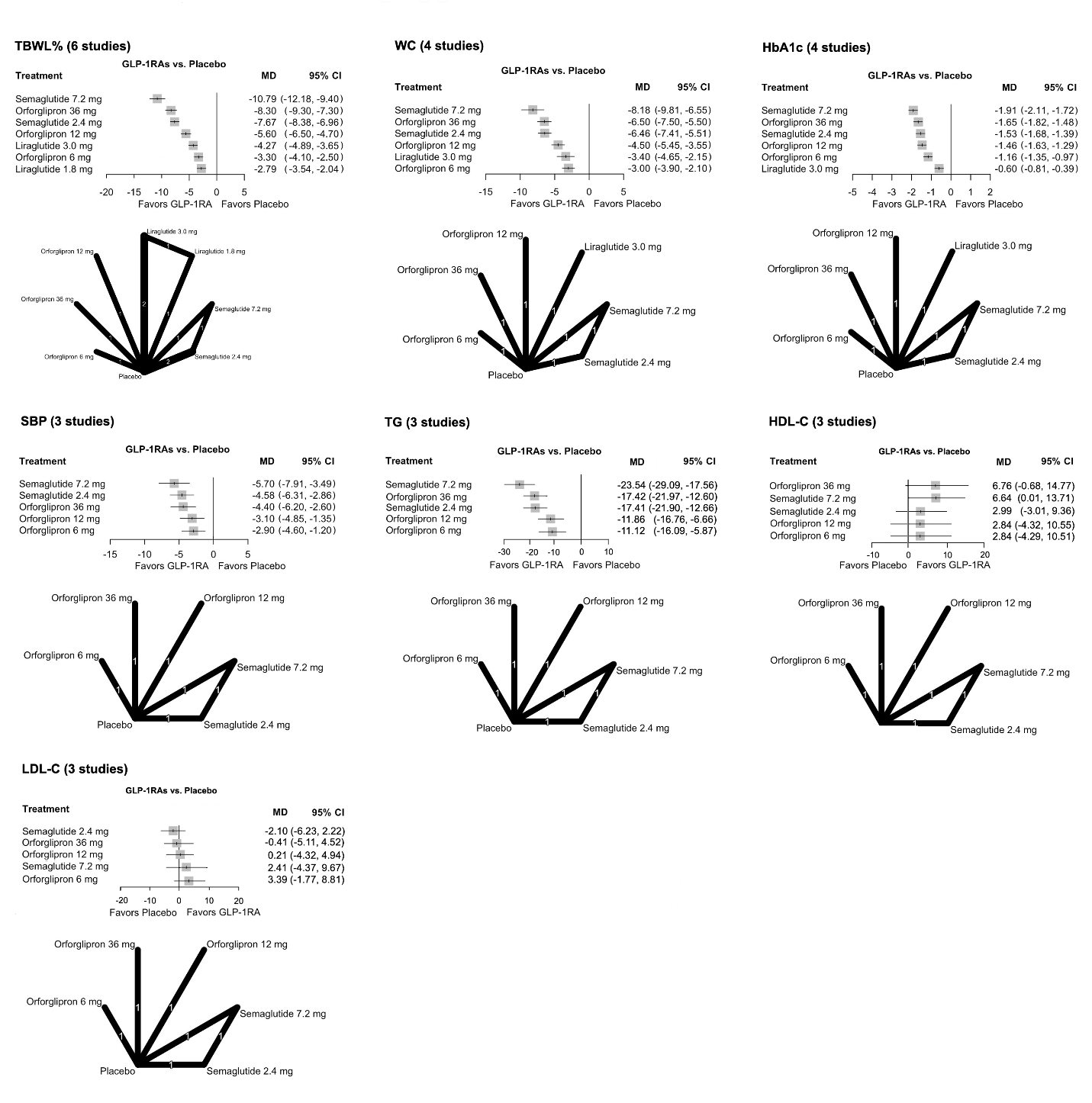


**Abbreviation:** HbA1c, haemoglobin A1c; HDL-C, high-density lipoprotein cholesterol; LDL-C, low-density lipoprotein cholesterol; SBP, systolic blood pressure; TBWL%, total body weight loss percentage; TG, triglyceride; WC, waist circumference.

**Note:** **Top panels:** Forest plots showing mean differences (MD) and 95% confidence intervals (CI) for each treatment versus placebo across seven cardiometabolic risk factors (TBWL%, WC, HbA1c, SBP, TG, HDL-C, and LDL-C).

**Bottom panels:** Network plots visualizing the evidence structure. Nodes represent specific treatment arms and lines (edges) connecting nodes indicate direct head-to-head clinical trials, with line thickness proportional to the number of studies informing each comparison (thicker lines indicate more direct evidence). Closed loops show comparisons supported by both direct and indirect evidence, enabling statistical assessment of network consistency.

**Figure S6.** Pairwise-origami plot summarizing multidimensional efficacy across seven cardiometabolic risk factors in adults with overweight or obesity with T2D


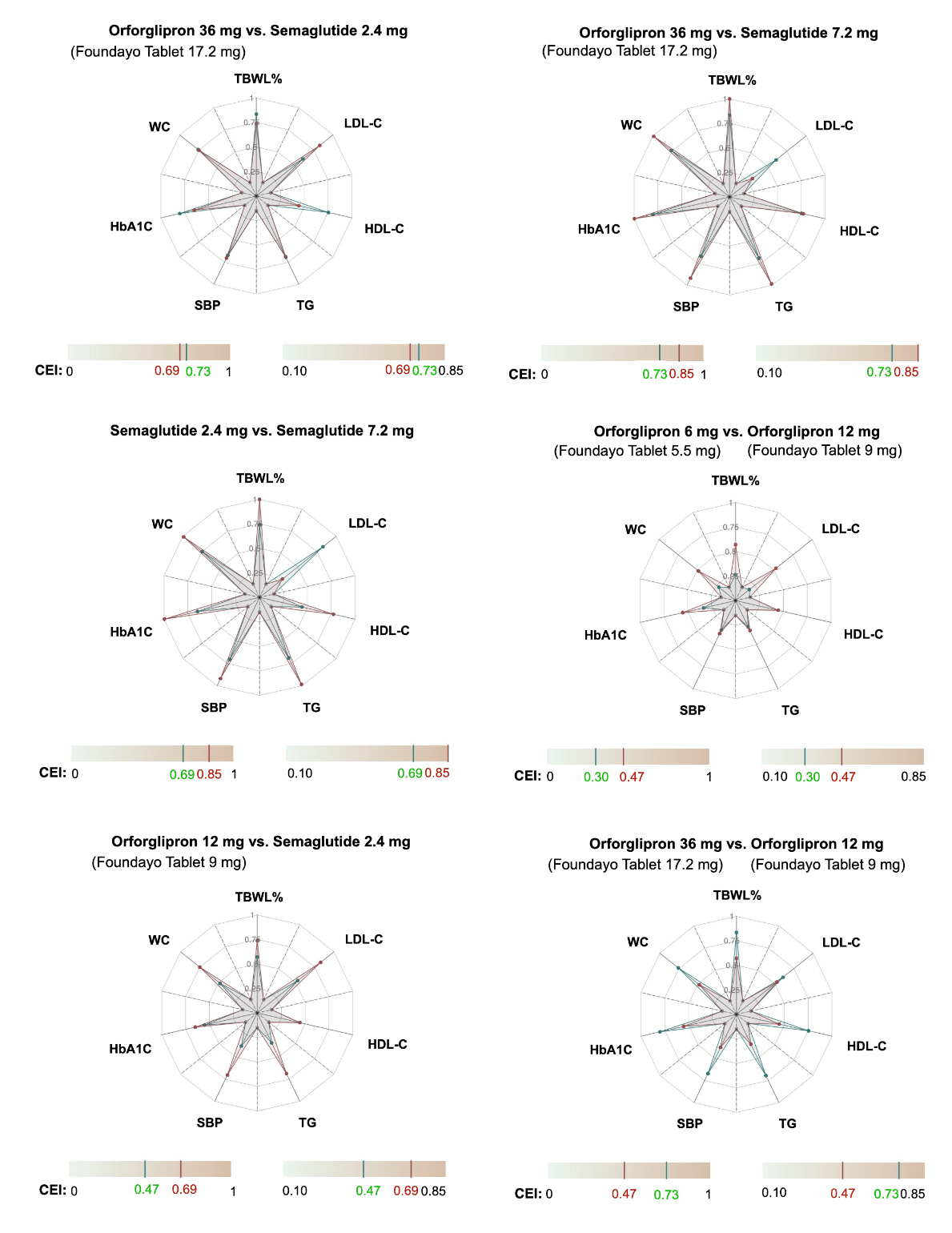


**Abbreviation:** HbA1c, haemoglobin A1c; HDL-C, high-density lipoprotein cholesterol; LDL-C, low-density lipoprotein cholesterol; SBP, systolic blood pressure; TBWL%, total body weight loss percentage; TG, triglyceride; WC, waist circumference.

**Note:** Origami plots visualize multidimensional cardiometabolic profiles using SUCRA rankings across seven outcomes (TBWL%, WC, HbA1c, SBP, TG, HDL-C, LDL-C). Each axis represents a SUCRA value, and the polygon area is normalized (0–1) to calculate the Cardiometabolic Efficacy Index (CEI), with larger areas indicating indicate more favourable overall profiles. In pairwise plots, the **green polygon** represents the primary treatment and the **red polygon** the comparator. The lower-left scale shows the normalized CEI (0–1), and the lower-right scale shows the absolute minimum and maximum polygon areas across all treatments in the network, with colour-coded values corresponding specifically to the two compared treatments.

**References**

1. Wharton S, Aronne LJ, Stefanski A, et al. Orforglipron, an Oral Small-Molecule GLP-1 Receptor Agonist for Obesity Treatment. *N Engl J Med*. 2025;393(18):1796-1806. doi:10.1056/NEJMoa2511774

2. Horn DB, Ryan DH, Kis SG, et al. Orforglipron, an oral small-molecule GLP-1 receptor agonist, for the treatment of obesity in people with type 2 diabetes (ATTAIN-2): a phase 3, double-blind, randomised, multicentre, placebo-controlled trial. *The Lancet*. 2025;406(10522):2927-2944. doi:10.1016/S0140-6736(25)02165-8

3. Davies MJ, Bergenstal R, Bode B, et al. Efficacy of Liraglutide for Weight Loss Among Patients With Type 2 Diabetes: The SCALE Diabetes Randomized Clinical Trial. *JAMA*. 2015;314(7):687-699. doi:10.1001/jama.2015.9676

4. Wadden TA, Tronieri JS, Sugimoto D, et al. Liraglutide 3.0 mg and Intensive Behavioral Therapy (IBT) for Obesity in Primary Care: The SCALE IBT Randomized Controlled Trial. *Obesity*. 2020;28(3):529-536. doi:10.1002/oby.22726

5. Garvey WT, Birkenfeld AL, Dicker D, et al. Efficacy and Safety of Liraglutide 3.0 mg in Individuals With Overweight or Obesity and Type 2 Diabetes Treated With Basal Insulin: The SCALE Insulin Randomized Controlled Trial. *Diabetes Care*. 2020;43(5):1085-1093. doi:10.2337/dc19-1745

6. Wilding JPH, Batterham RL, Calanna S, et al. Once-Weekly Semaglutide in Adults with Overweight or Obesity. *N Engl J Med*. 2021;384(11):989-1002. doi:10.1056/NEJMoa2032183

7. Davies M, Færch L, Jeppesen OK, et al. Semaglutide 2·4 mg once a week in adults with overweight or obesity, and type 2 diabetes (STEP 2): a randomised, double-blind, double-dummy, placebo-controlled, phase 3 trial. *The Lancet*. 2021;397(10278):971-984. doi:10.1016/S0140-6736(21)00213-0

8. Wadden TA, Bailey TS, Billings LK, et al. Effect of Subcutaneous Semaglutide vs Placebo as an Adjunct to Intensive Behavioral Therapy on Body Weight in Adults With Overweight or Obesity: The STEP 3 Randomized Clinical Trial. *JAMA*. 2021;325(14):1403-1413. doi:10.1001/jama.2021.1831

9. Garvey WT, Batterham RL, Bhatta M, et al. Two-year effects of semaglutide in adults with overweight or obesity: the STEP 5 trial. *Nat Med*. 2022;28(10):2083-2091. doi:10.1038/s41591-022-02026-4

10. Kadowaki T, Isendahl J, Khalid U, et al. Semaglutide once a week in adults with overweight or obesity, with or without type 2 diabetes in an east Asian population (STEP 6): a randomised, double-blind, double-dummy, placebo-controlled, phase 3a trial. *Lancet Diabetes Endocrinol*. 2022;10(3):193-206. doi:10.1016/S2213-8587(22)00008-0

11. Mu Y, Bao X, Eliaschewitz FG, et al. Efficacy and safety of once weekly semaglutide 2·4 mg for weight management in a predominantly east Asian population with overweight or obesity (STEP 7): a double-blind, multicentre, randomised controlled trial. *Lancet Diabetes Endocrinol*. 2024;12(3):184-195. doi:10.1016/S2213-8587(23)00388-1

12. Rubino DM, Greenway FL, Khalid U, et al. Effect of Weekly Subcutaneous Semaglutide vs Daily Liraglutide on Body Weight in Adults With Overweight or Obesity Without Diabetes: The STEP 8 Randomized Clinical Trial. *JAMA*. 2022;327(2):138-150. doi:10.1001/jama.2021.23619

13. Bliddal H, Bays H, Czernichow S, et al. Once-Weekly Semaglutide in Persons with Obesity and Knee Osteoarthritis. *N Engl J Med*. 2024;391(17):1573-1583. doi:10.1056/NEJMoa2403664

14. McGowan BM, Bruun JM, Capehorn M, et al. Efficacy and safety of once-weekly semaglutide 2·4 mg versus placebo in people with obesity and prediabetes (STEP 10): a randomised, double-blind, placebo-controlled, multicentre phase 3 trial. *Lancet Diabetes Endocrinol*. 2024;12(9):631-642. doi:10.1016/S2213-8587(24)00182-7

15. Wharton S, Freitas P, Hjelmesæth J, et al. Once-weekly semaglutide 7·2 mg in adults with obesity (STEP UP): a randomised, controlled, phase 3b trial. *Lancet Diabetes Endocrinol*. 2025;13(11):949-963. doi:10.1016/S2213-8587(25)00226-8

16. Lingvay I, Bergenheim SJ, Buse JB, et al. Once-weekly semaglutide 7·2 mg in adults with obesity and type 2 diabetes (STEP UP T2D): a randomised, controlled, phase 3b trial. *Lancet Diabetes Endocrinol*. 2025;13(11):935-948. doi:10.1016/S2213-8587(25)00225-6

17. Kosiborod MN, Abildstrøm SZ, Borlaug BA, et al. Semaglutide in Patients with Heart Failure with Preserved Ejection Fraction and Obesity. *N Engl J Med*. 2023;389(12):1069-1084. doi:10.1056/NEJMoa2306963

18. Kosiborod MN, Petrie MC, Borlaug BA, et al. Semaglutide in Patients with Obesity-Related Heart Failure and Type 2 Diabetes. *N Engl J Med*. 2024;390(15):1394-1407. doi:10.1056/NEJMoa2313917

19. Wharton S, Lingvay I, Bogdanski P, et al. Oral Semaglutide at a Dose of 25 mg in Adults with Overweight or Obesity. *N Engl J Med*. 2025;393(11):1077-1087. doi:10.1056/NEJMoa2500969
